# Heterologous Immunity between Adenoviruses and Hepatitis C Virus: A New Paradigm in HCV Immunity and Vaccines

**DOI:** 10.1371/journal.pone.0146404

**Published:** 2016-01-11

**Authors:** Shakti Singh, Satish Vedi, Subodh Kumar Samrat, Wen Li, Rakesh Kumar, Babita Agrawal

**Affiliations:** 1 Department of Surgery, Faculty of Medicine and Dentistry, University of Alberta, Edmonton, Alberta, Canada; 2 Department of Laboratory Medicine and Pathology, Faculty of Medicine and Dentistry, University of Alberta, Edmonton, Alberta, Canada; University of Cape Town, SOUTH AFRICA

## Abstract

Adenoviruses (Ad) are commonly used as vectors for gene therapy and/or vaccine delivery. Recombinant Ad vectors are being tested as vaccines for many pathogens. We have made a surprising observation that peptides derived from various hepatitis C virus (HCV) antigens contain extensive regions of homology with multiple adenovirus proteins, and conclusively demonstrate that adenovirus vector can induce robust, heterologous cellular and humoral immune responses against multiple HCV antigens. Intriguingly, the induction of this cross-reactive immunity leads to significant reduction of viral loads in a recombinant vaccinia-HCV virus infected mouse model, supporting their role in antiviral immunity against HCV. Healthy human subjects with Ad-specific pre-existing immunity demonstrated cross-reactive cellular and humoral immune responses against multiple HCV antigens. These findings reveal the potential of a previously uncharacterized property of natural human adenovirus infection to dictate, modulate and/or alter the course of HCV infection upon exposure. This intrinsic property of adenovirus vectors to cross-prime HCV immunity can also be exploited to develop a prophylactic and/or therapeutic vaccine against HCV.

## Introduction

Chronic infection with hepatitis C virus (HCV) is a serious global health problem. It affects ~170 million people worldwide and can lead to liver cirrhosis, hepatocellular carcinoma and end-stage liver diseases [1–3]. Current treatment of chronic HCV infection is limited to combination drug therapies, which are highly expensive, have serious side effects and have variable success rates in different viral genotypes and patient populations [4]. Development of vaccines to prevent and/or cure HCV infection is of paramount importance.

Natural protection from infection is often used to model strategies to develop new vaccines. The immune mechanisms and correlates of viral clearance and protection from HCV infection have been studied extensively, but they still remain unclear [5–7]. A number of studies have correlated the natural clearance of HCV infection with the appearance of vigorous and multi-specific CD8^+^ and sustained CD4^+^ T cell responses [8–10]. In contrast, development of chronic infection is correlated with weak or dysfunctional cellular immune responses [7, 10, 11]. A recent meta-analysis of studies published to date with chimpanzee model concluded that humoral immune responses also play a determining role in protection from chronic HCV infection [12–14]. Further, recent studies support a role of antibodies beyond neutralization of viral particles; extending their function to targeting intracellular pathogens [15]. Therefore, it can be envisioned that a successful prophylactic and/or therapeutic vaccine strategy would encompass the generation of both cellular and humoral responses against multiple antigens of HCV.

Cases of chronic HCV infection have been reported where patients spontaneously clear the infection due to re-activation of functional cellular immune responses [16]. However, the exact mechanism of this spontaneous reversal of cellular immune responses, from exhausted and/or dysfunctional to functional, remains to be elucidated. In several studies, HCV-specific T cells have been expanded from the peripheral blood T-cell populations in individuals previously not infected with HCV [17, 18]. Among the HCV exposed individuals, 20–30% clear the virus after acute infection due to induction of strong multi-specific cellular immune responses [10, 19, 20]. However, it is not known if pre-existing heterologous immunity has any role in the HCV clearance after infection. Influenza A virus infection has been shown to induce narrow heterologous cellular immune response against a single epitope from HCV NS3 antigen [18].

Adenoviruses (Ad) are non-enveloped, icosahedral viruses containing a double-stranded DNA genome of ~26–48 Kbp [21]. They belong to a diverse family (>50 serotypes) of DNA viruses called *adenoviridae*, infect ocular, respiratory or gastro-intestinal epithelium in a diverse range of hosts [21] and induce different levels of immunity [22, 23]. They have gained immense recognition as a vaccine antigen delivery vehicle since their initial use in gene therapy [24], and have also proven to be safe and efficient vaccine vectors for eliciting protective immune responses against the transgene antigen in many animal and human clinical studies [25, 26]. A chimpanzee-derived adenovirus, expressing HCV antigens NS3-NS5, is currently in clinical trials [27].

We have been studying recombinant adenoviruses expressing various individual HCV antigens for their ability to modulate and induce HCV-specific immunity in an *in vitro* human cell culture system and *in vivo* in mice [28–32]. During our studies, we observed that immunization of mice with control, non-recombinant adenovirus vector (Ad vector) not expressing HCV antigens, also induced HCV antigens-specific T cell responses. To address this puzzling and unexpected observation, we aligned peptide sequences from different HCV proteins with various adenoviral proteins (Ad proteins) to determine the levels of homology between the proteins of the two viruses. Intriguingly, we discovered varying degrees of homologies (25–53%) between multiple HCV peptide sequences from core, F, NS3, NS4 and NS5 proteins and a number of Ad proteins (Table 1 and S1 Table). In this study, we conclusively demonstrate that immunization of mice with Ad vector not expressing exogenous HCV antigen(s), induces robust cross-reactive humoral and cellular immune responses against multiple HCV antigens. Further, reduction in viral titers in mice infected with vaccinia virus expressing recombinant HCV antigens as a surrogate model corroborated the relevance of cross-reactive immune responses to antiviral immunity. Intriguingly, normal healthy human donors with no known history of HCV infection but seropositive for Ad-specific IgG, demonstrated the presence of cross-reactive antibodies and T cell responses against multiple HCV antigens. Our study is the first to demonstrate heterologous and protective immunity induced by Ad vector against HCV. These studies have significant implications in the design of prophylactic and therapeutic vaccines against HCV. In addition, since recombinant adenovirus vectors are at the forefront as candidate vaccines for several different pathogens including HCV, Ebola virus, Plasmodium, mycobacteria, influenza virus, among others, their widespread use as vaccines could significantly impact the prevalence and natural course of HCV infection.

**Table 1:** Summary of HCV peptides showing homology to adenovirus (Ad) proteins.

No.	HCV proteins	No. of HCV peptides tested	No. of Ad proteins tested	Number of peptides showing homology (% Homology between HCV peptide and Ad protein sequences)					
				(25.00–30.00)	(30.11–35.00)	(35.11–40.00)	(40.11–45.00)	(45.11–50.00)	(>50.00)
**1**	**Core**	45	27	45	43	27	0	7	1
**2**	**F**	16	27	16	15	4	0	3	1
**3**	**NS3**	11	27	10	8	8	1	1	0
**4**	**NS4**	20	27	15	7	2	0	0	0
**5**	**NS5a**	29	27	29	10	7	2	0	0
**6**	**NS5b**	39	27	39	7	4	1	0	0

## Results

### Peptide sequences derived from HCV antigens exhibit varying degrees of homology with different adenoviral (Ad) vector proteins

We aligned the amino acid (aa) sequences of peptides (15–20 aa long with 5 aa overlaps) derived from various HCV antigens (core, F, E1, E2, P7, NS2, NS3, NS4a, NS4b, NS5a and NS5b) of genotype 1a with different Ad proteins using the bioinformatics software ClustalW to determine homology scores. Table 1 summarizes the range of homologies between HCV-derived peptides and Ad proteins, and the number of HCV peptides and Ad proteins that show these homologies. Intriguingly, almost all of the compared Ad proteins (27) (S1 Table) showed homology (scores ranging between 25–40) with several peptides from HCV antigens core, F, NS3, NS4, NS5a and NS5b (S1–S6 Datasets). The maximum homology scores shown by HCV peptides was core (45–50), F (45–50), NS3 (45–50), NS4 (35–40), NS5a (40–45) and NS5b (40–45). Similar homologies were found with ChAd25 sequences. HCV antigens E1, E2, P7 and NS2 showed low homology (<25%) with the lowest number of Ad proteins (data not shown), therefore these were not tested for cross-reactive immune responses induced by Ad vector.

### DNA isolated from Ad vector does not contain HCV genes

**Fig 1:**
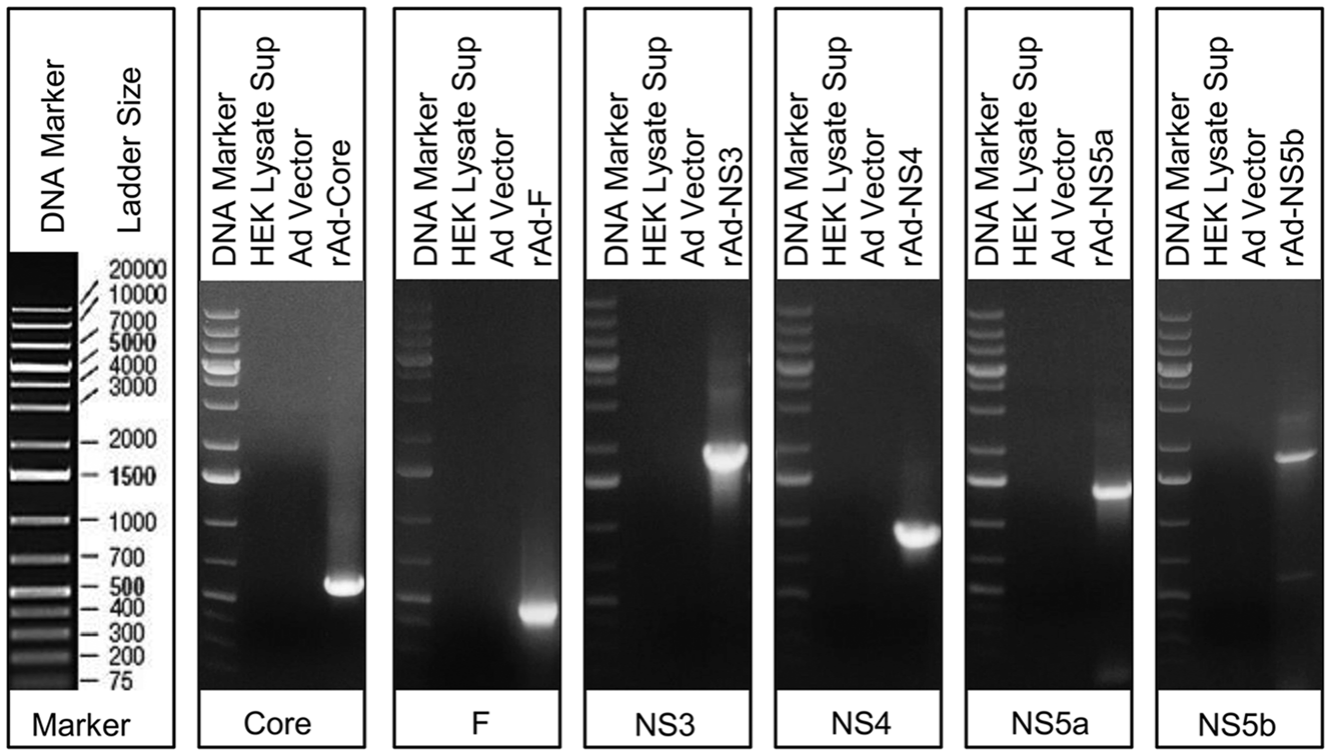
Characterization of Ad vector stock by PCR. HCV genes core, F, NS3, NS4, NS5a or NS5b are not amplified. First panel shows the DNA ladder, followed by agarose gel electrophoresis of PCR products obtained with HCV core, F, NS3, NS4, NS5a and NS5b specific primers. HEK lysate supernatant and rAd-HCV vectors were used as negative and positive controls. Data are representative of 2–3 repeated experiments.

To exclude the possibility of contamination of the non-recombinant Ad vector stock with HCV transgene, we set up PCR with purified Ad DNA using HCV-specific primers (listed in S2 Table). None of the primer sets specific for core, F, NS3, NS4, NS5a or NS5b genes of HCV were able to amplify HCV gene products from non-recombinant Ad vector DNA template (Fig 1). Uninfected HEK cell lysate and recombinant adenoviral (rAd) vectors containing individual HCV genes were used as negative and positive controls, respectively.

### Immunization of mice with Ad vector induces cross-reactive cellular and humoral immune responses against multiple HCV antigens

**Fig 2:**
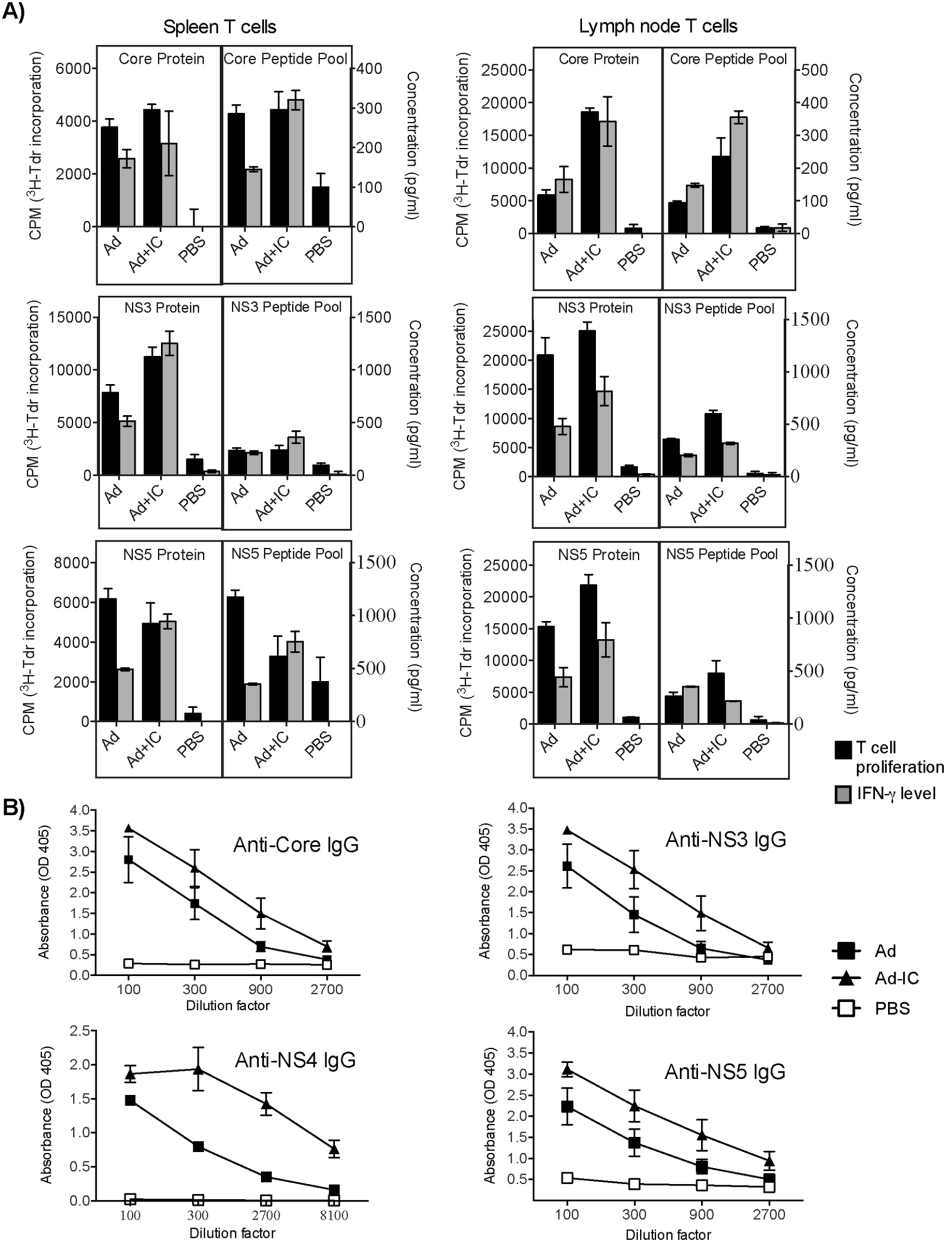
Identification of cross-reactive cellular and humoral immune responses in mice immunized with Ad vector (with or without poly I:C adjuvant). **(A)** Proliferation and IFN-γ production in spleen and lymph node T cells upon *ex vivo* stimulation with HCV antigens, or a pool of 5 peptides showing high homology with Ad proteins (see text for details). **(B)** Cross-reactive antibody response against HCV antigens. Data are presented as mean±standard deviation of 3–4 replicates, and represent more than three independent experiments.

The significant homologies of HCV derived peptides with Ad proteins sequences indicated that adenoviruses might induce cross-reactive immunity against HCV antigens. To test this, female C57bl/6 mice (n = 5/group) were immunized intramuscularly twice (at 14 days interval) with 2x10^7^ PFU/mouse Ad vector, Ad vector + poly I:C or PBS. The proliferation and IFN-γ production from spleen and lymph node T cells were determined in response to HCV core, NS3 and NS5 protein antigens and pools of their selected peptides (listed in S1–S6 Datasets) (core peptides #: 5, 14, 16, 17 & 27; NS3 peptides #: 2, 5, 6, 8 &10; NS5a peptides #: 6, 24; NS5b peptides #: 5, 19, 27) (Fig 2A). Both spleen and inguinal lymph node T cells from Ad vector immunized mice demonstrated high HCV antigens (core, NS3 and NS5)-dependent proliferation and IFN-γ production, which was further increased by co-immunization of Ad vector with poly I:C adjuvant. Cross-reactive proliferation and IFN-γ responses against selected individual peptides of core, F, NS3, NS4 and NS5 antigens are shown in supplementary information (S1A–S1F and S2 Figs). T cell proliferation and IFN-γ responses were also examined against synthetic peptides from HCV antigens NS3, NS4 and NS5 showing low (<20%) or no homology, and responses were not obtained above background (data not shown).

We also examined the induction of cross-reactive IgG antibodies against HCV protein antigens (core, NS3, NS4 and NS5) in the serum samples obtained from immunized mice (Fig 2B). The Ad vector induced significant amounts of cross-reactive IgG against all of the HCV antigens. Co-administration of TLR agonist poly I:C further enhanced the levels of cross-reactive antibodies. PBS-immunized mice did not show IgG binding to any of the HCV antigens. Also, Ad vector immunized mice did not show antibody binding to control protein antigen rhSOD (data not shown).

**Fig 3:**
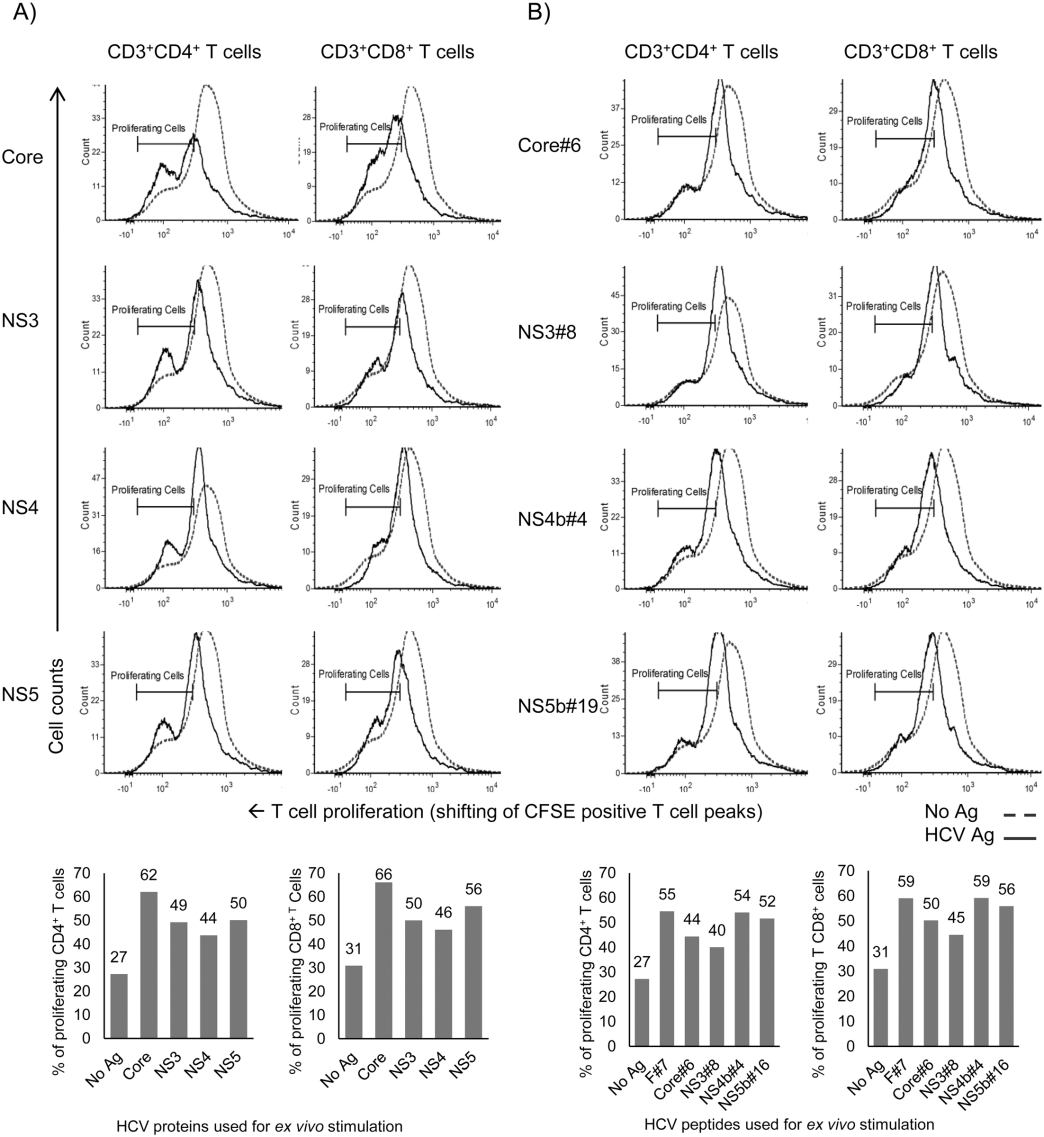
Cross-reactive CD4^+^ and CD8^+^ T cells obtained from Ad vector immunized mice proliferate in HCV antigens-dependent manner. Splenocytes obtained from Ad vector immunized mice were stimulated *ex vivo* with various recombinant HCV antigens (core, NS3, NS4 and NS5) or their selected respective peptides (at 5 μg/ml each), and analyzed by flow cytometry. Proliferation of CD4^+^ and CD8^+^ T cells stimulated *ex vivo* with: **(A)** HCV proteins; **(B)** Representative peptides derived from HCV proteins using the CFSE-based assay (loss of CFSE due to cell division represented by shift of peak of CFSE^+^ T cells towards left). Data are obtained from a pool (n = 5) of spleen cells and are representative of two independent experiments.

To confirm that the high proliferation responses observed in the ^3^H-Tdr assay are due to actual proliferation of cross-reactive HCV-specific CD4^+^ and CD8^+^ T cells, we performed a CFSE dilution assay along with staining for CD3, CD4 and CD8 markers. Splenocytes obtained from Ad vector immunized mice were stimulated *ex vivo* with HCV protein antigens (core, NS3, NS4 and NS5) (Fig 3A), or selected representative peptides from these proteins (Fig 3B). Intriguingly, the stimulated splenocytes exhibited remarkable antigen-dependent proliferation of both cross-reactive CD3^+^CD4^+^ and CD3^+^CD8^+^ T cells.

**Fig 4:**
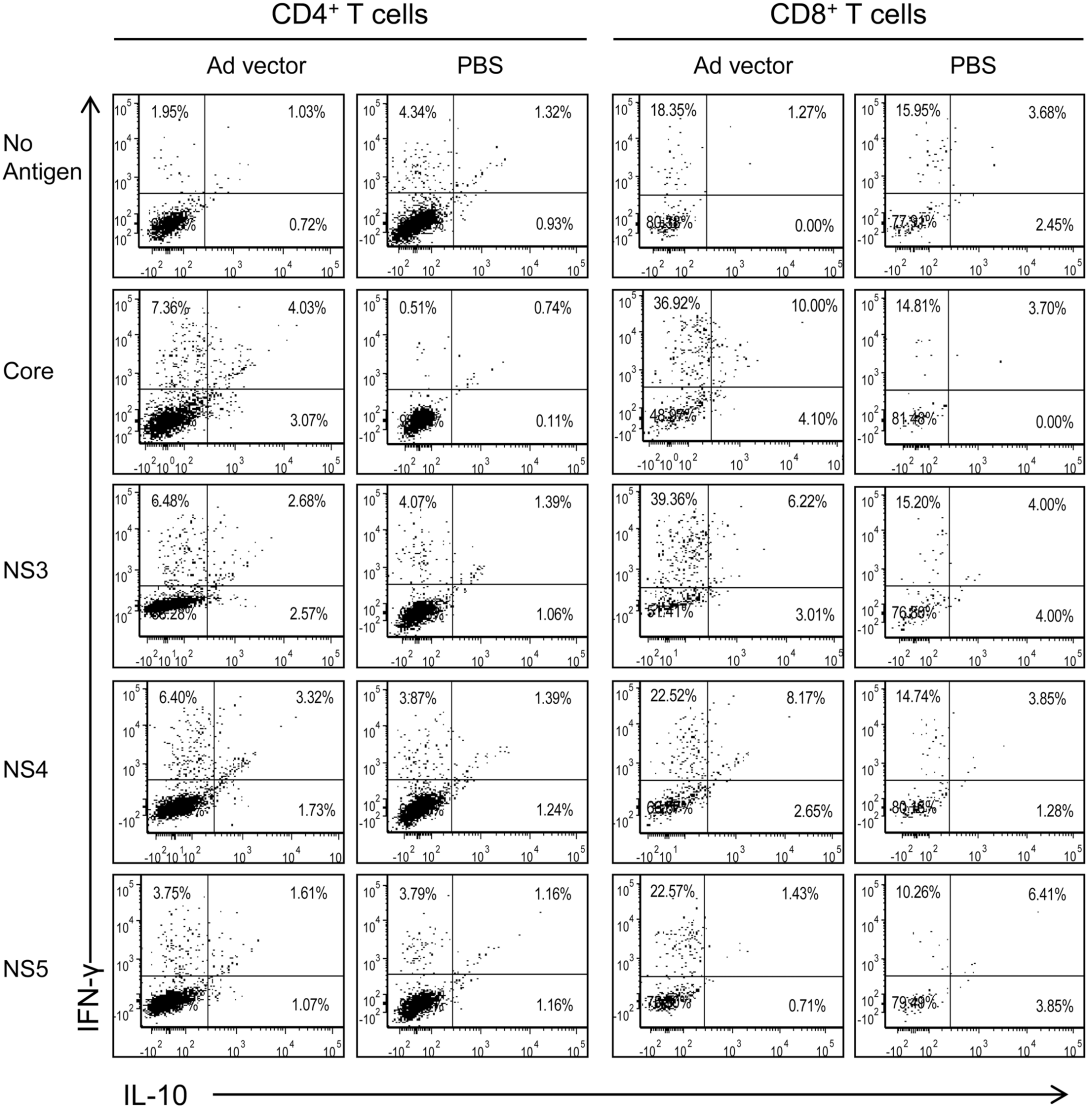
Cross-reactive CD4^+^ and CD8^+^ T cells obtained from Ad vector immunized mice produce cytokines upon *ex vivo* stimulation with various HCV proteins. Splenocytes obtained from Ad vector immunized mice were cultured with HCV core, NS3, NS4 or NS5 antigens at 5 μg/ml, and analyzed after 5 days for intracellular IFN-γ and IL-10 expression profile of CD4^+^ and CD8^+^ T cells by flow cytometry. Data are obtained from a pool (n = 5) of spleen cells and are representative of two independent experiments.

**Fig 5:**
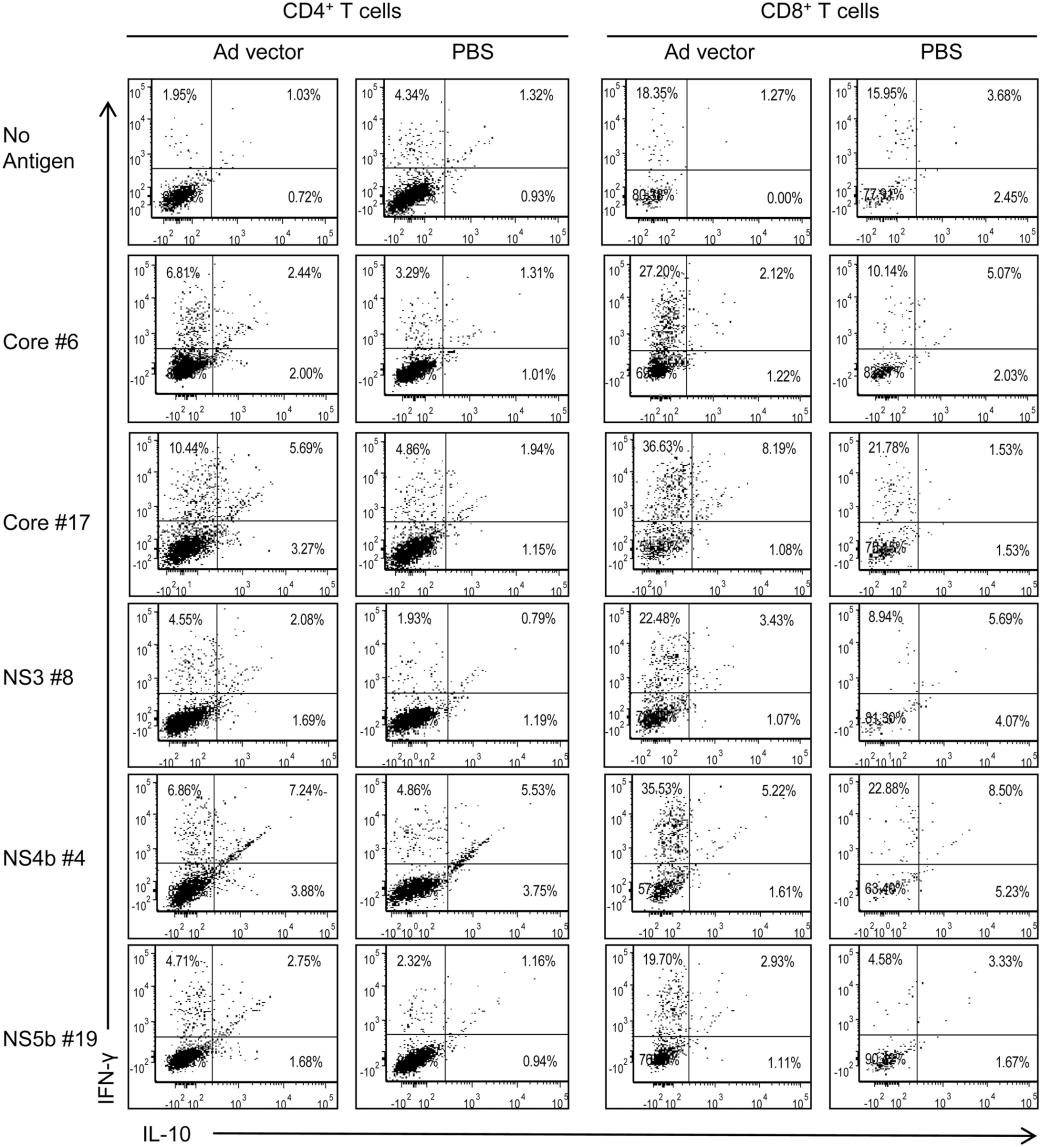
Cross-reactive CD4^+^ and CD8^+^ T cells obtained from Ad vector immunized mice produce cytokines upon *ex vivo* stimulation with HCV peptides. Splenocytes obtained from Ad vector immunized mice were cultured with representative peptides derived from HCV core, NS3, NS4 or NS5 at 5 μg/ml each, and analyzed after 5 days for intracellular IFN-γ and IL-10 expression profile of CD4^+^ and CD8^+^ T cells by flow cytometry. Data are obtained from a pool (n = 5) of spleen cells and are representative of two independent experiments.

To demonstrate that the cytokines produced in culture supernatants are from CD4^+^ and CD8^+^ T cells, we performed intracellular cytokine expression analyses of splenocytes obtained from Ad vector immunized mice and stimulated *ex vivo* with HCV core, NS3, NS4 and NS5 protein antigens (Fig 4) and their selected peptides (Fig 5). Splenocytes from mice immunized with PBS and cultured with various HCV peptide or protein antigens, and splenocytes from mice immunized with Ad vector and incubated with medium were used as negative controls (Figs 4 & 5). Both CD4^+^ and CD8^+^ T cells from Ad immunized mice showed increased expression of IFN-γ and IL-10 simultaneously upon stimulation with HCV proteins when compared to PBS immunized mice (Fig 4). We noted that HCV core-stimulated CD4^+^ and CD8^+^ T cells showed a high frequency of IL-10 expressing cells compared to other HCV antigens. However, IFN-γ producing CD4^+^ and CD8^+^ T cells were high against all HCV antigens except HCV NS5 where only IFN-γ producing CD8^+^ T cells were increased (Fig 4). Upon stimulation with HCV core, NS3, NS4 and NS5 peptides, cross-reactive CD4^+^ T cells showed enhanced IFN-γ and IL-10 expression in comparison to the PBS group (Fig 5). Further, IFN-γ-producing CD8^+^ T cells were notably high with all the peptides used for *in vitro* stimulation (Fig 5). However, the frequency of CD8^+^ T cells, which express IL-10 or both IFN-γ and IL-10, were not increased with any of the selected peptides except HCV core peptide #17, which also showed increased frequency of double positive (both IFN-γ and IL-10) CD4^+^ T cells (Fig 5).

### Cross-reactive effector cells induced upon immunization with Ad vector exert cytotoxicity to target cells loaded with HCV-derived peptides

**Fig 6:**
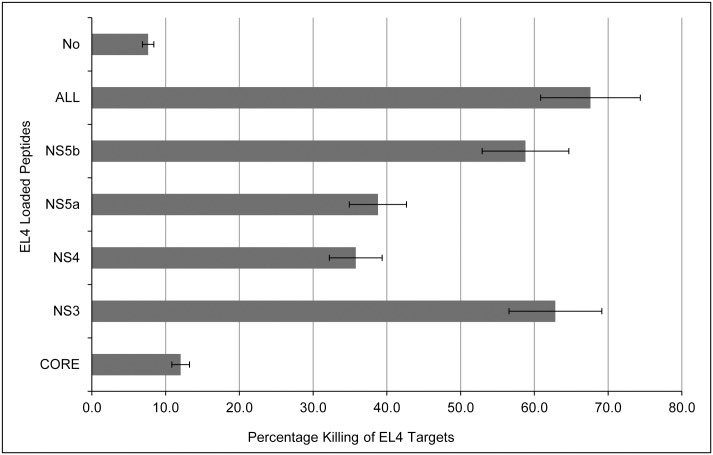
Cytotoxic killing of target cells loaded with HCV antigens-derived peptides, by splenocytes obtained from Ad vector immunized mice. Splenocytes harvested from Ad vector immunized mice, were stimulated *in vitro* with the HCV protein antigens core, NS3, NS4 and NS5 at 5 μg/ml concentrations for 4 days. The target EL4 cells were incubated with corresponding HCV peptides each at 1 μg/ml concentration (core peptides #: 2, 14, 17, 25, 27, 28, 32; NS3 peptides #: 8, 10; NS4 peptides #: 3, 4, 8; NS5a peptides #: 1, 2, 16, 20 and NS5b peptides: 5, 19, 23, 39; or All: a mixture of the above peptides from core, NS3, NS4 and NS5) and peptide-loaded EL4 cells were cultured with effectors at 10:1 (effectors: target) ratio for 4–5 hours. Empty (no peptide loaded) EL4 targets were used as a negative control. CFSE-labeled live targets were quantified by flow cytometry, and % killed targets were calculated using the formula: % Killing = [(Average live cells in PBS control − live cells in immunized group) /Average live cells in PBS control] × 100). Data shown are mean±SD and are representative of three independent experiments.

The cytotoxic activity of the cross-reactive effector cells obtained from the spleens of Ad vector immunized mice and stimulated *in vitro* with HCV protein antigens (core, NS3, NS4, NS5 or polyprotein) was examined against EL4 target cells loaded with pools of respective HCV antigen peptides. Different sets of CFSE-stained EL4 targets were prepared by loading them with pools of HCV antigen peptides: core (peptides # 2, 14, 17, 25, 27, 28, 32), NS3 (peptides # 8, 10), NS4 (peptides # 3, 4, 8), NS5 (peptides # NS5a: 1, 2, 16, 20 and NS5b: 5, 19, 23, 39) (listed in S1–S6 Datasets), all of the above peptides (ALL) or no peptide loaded (No). These different EL4 targets were cultured with corresponding splenocytes stimulated with HCV protein antigens. The results showed that splenocytes induced after Ad vector immunization had potent cytotoxic effector function, in an antigen-specific manner. Interestingly, *ex vivo* stimulation of cross-reactive effectors with core antigen showed the least antigen-specific cytotoxic activity compared to other HCV antigens (Fig 6).

### Role of the HCV-specific cross-reactive immune responses induced by Ad vector in reducing viral loads in Vaccinia-HCV challenged mice

**Fig 7:**
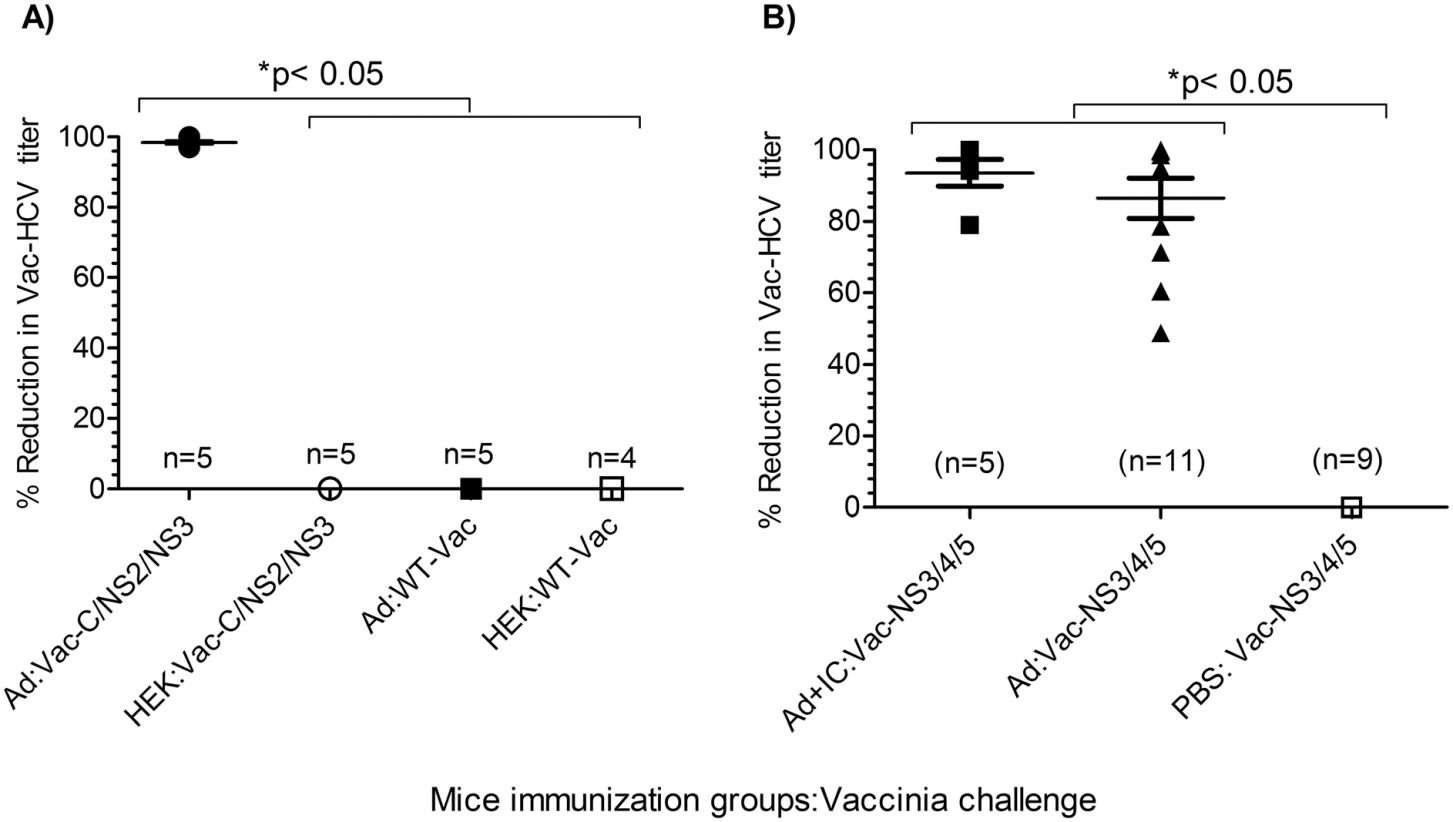
Immunization of mice with Ad vector leads to reduced titer of Vaccinia-HCV chimeric virus. Mice immunized twice intramuscularly with Ad vector (with or without poly I:C), PBS, or HEK cell lysate, were challenged 8 days after the second immunization with wild-type Vaccinia (WT-Vac) or chimeric Vaccinia-HCV. Ovaries were harvested 5 days after challenge and viral loads in each mouse were determined by plaque-forming assay using TK-1 cells. **(A)** Challenge with HCV core-NS2-NS3 (Vac-C/NS2/NS3) or wild type vaccinia (WT-Vac). **(B)** Challenge with Vaccinia-HCV NS3-NS4-NS5 (Vac-NS3/4/5). Data are presented as mean ± standard deviation of % reduction in viral titer compared to corresponding unimmunized control group, and statistical comparison was done by two-tailed *t*-test (p<0.05 was considered significant).

To demonstrate the antiviral potential of Ad vector induced cross-reactive immune responses against HCV, a surrogate Vac-HCV infection model [32] was used. Although this is not a direct model of HCV infection, it is a valuable model to assess the ability of the induced immunity to kill host targets expressing HCV antigens. Reduction in Vac-HCV viral titers in this model is a critical parameter for the antiviral response against HCV. C57bl/6 mice, after two immunizations with Ad vector with or without poly I:C as adjuvant, were challenged with wild-type vaccinia (WT-Vac), or Vaccinia-HCV chimeric viruses (Vac-HCV core-NS3 or Vac-HCV NS3-NS4-NS5). Five days after challenge, viral loads were determined in ovaries in individual mice (Fig 7). The mice immunized with Ad vector in the presence or absence of poly I:C had significantly (p <0.05) reduced Vac-HCV (core-NS2-NS3 or NS3-NS4-NS5) viral loads in comparison to PBS or HEK cell lysate-immunized mice (Fig 7A and 7B). In contrast, viral titers in WT-Vaccinia challenged mice were not significantly (p = 0.8472) different in HEK lysate or Ad vector immunized mice (Fig 7A). These results provide strong evidence that the Ad vector induced cross-reactive immunity specifically targets HCV antigens.

### Humans with pre-existing Ad immunity demonstrate cross-reactive humoral and cellular immune responses against HCV antigens

**Fig 8:**
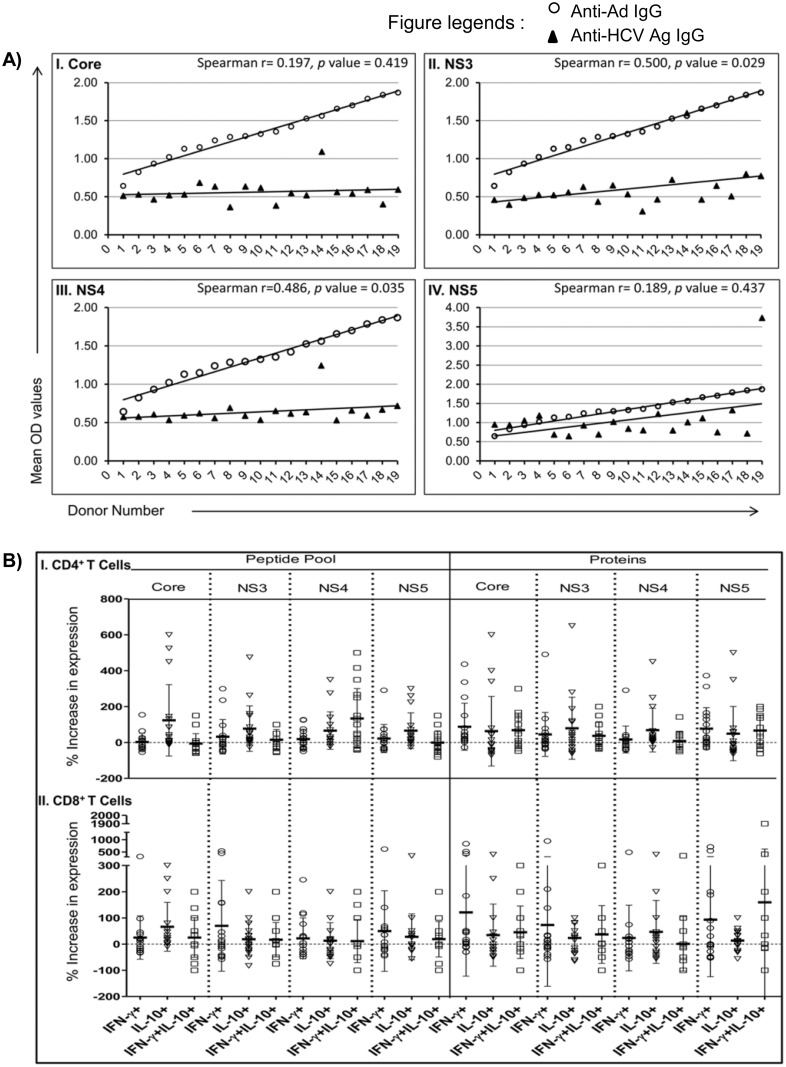
Healthy humans with pre-existing Ad immunity show HCV-specific cross-reactive immune responses. **(A)** Correlation of anti-Ad IgG with cross-reactive IgG against different HCV antigens. OD values for Ad-specific IgG (open circles) from 19 healthy donors were arranged in ascending order and matched with corresponding OD values for IgG against HCV core, NS3, NS4 or NS5 antigens (solid triangles). The Spearman coefficients were calculated for the data points that did not show normal distributions. **(B)** Expression of IFN-γ and IL-10 in CD4^+^ and CD8^+^ T cells upon *ex vivo* stimulation with HCV peptide pools or protein antigens. Data are shown as the percent increase in expression of IFN-γ, IL-10 and IFN-γ/IL-10 in an HCV antigens-dependent manner compared to controls (media). Data for each donor and data points from all donors (n = 17, two donors in the cohort did not provide enough blood samples to do cellular assays) are plotted in stacked dot plots as mean±SD.

To understand the induction of cross-reactive immunity in HCV-naive humans, we examined Ad-specific and HCV-specific cross-reactive IgGs in healthy human blood donors (n = 19) (Fig 8A). There was a significant direct correlation between the presence of Ad-specific IgG with HCV NS3 (Spearman r = 0.500, *p* value = 0.029) and NS4 (Spearman r = 0.486, *p* value = 0.035) (Fig 8AII and 8AIII). We also detected cross-reactive IgG against HCV core and NS5 antigens; however the correlations between these antibodies with the Ad hexon-specific IgG (Fig 8AI and 8AIV) were not statistically significant.

Next, as an indication of cross-reactive cellular immune response, we determined the intracellular expression of IFN-γ and IL-10 in CD4^+^ and CD8^+^ T cell subsets upon *ex vivo* stimulation of PBMCs with HCV peptide pool or protein antigens for 4 days. Interestingly, both CD4^+^ and CD8^+^ T cells from these healthy donors (n = 17, two donors did not provide enough blood samples to isolate PBMCs) demonstrated induction of HCV antigens-specific IFN-γ and/or IL-10 (Fig 8B). Overall, there was a propensity of IL-10 induction by CD4^+^ T cells upon stimulation with peptide pools from all four HCV antigens and NS3 and NS4 protein antigens. However, a number of donors showed increased IFN-γ and IFN-γ/IL-10 expression in both CD4^+^ and CD8^+^ T cells upon stimulation with HCV antigens (Fig 8B).

## Discussion

In the present study, we demonstrate for the first time that Ad vector has the intrinsic ability to induce broad and robust cellular and humoral immune responses against a number of HCV structural and non-structural antigens. The *in silico* sequence alignment studies (Table 1 and S1 Table, S1–S6 Datasets) exhibited an interesting and unexpected pattern of homologies in the amino acid sequences between HCV and Ad protein sequences. Homology ranged from 25–53% (homologies below 25% are not reported here), with HCV core peptide epitopes showing the greatest amino acid homology to the largest number of Ad proteins. Further, HCV frame-shift protein (F), NS3, NS4, and NS5 also showed significant levels of homology with Ad proteins.

Our results, presented in Figs 2–5 (and also S1 and S2 Figs), provide conclusive evidence that Ad vector induced significant levels of cross-reactive immune responses against HCV antigens. These immune responses are mediated by both CD4^+^ and CD8^+^ T cells, and cover a broad range of HCV peptide epitopes. We also showed that heterologous effector cells, induced by Ad vector immunization, possess strong cytotoxic effector function (Fig 6).

Heterologous immunity is the term used when immune responses induced by one virus confers immunity to another unrelated virus and/or its antigens. Indeed, there have been reports showing induction of cross-reactive T cell responses among unrelated viruses [18, 33–35] Adenovirus and hepatitis C virus are evolutionarily unrelated. They differ in host cell specificity and contain genomes of dsDNA and RNA, respectively. However, the induction of heterologous immunity does not require the entire T cell epitope to be homologous [36]. This immunity can result from similarities in amino acid sequences in small stretches of peptides and/or promiscuity of T cell receptors (TCR) [36–39]. It has been shown that only 2–4 amino acids in the T cell epitopes actively participate in interaction with the TCR, and stronger associations in peptide-MHC and TCR is provided by co-stimulatory molecules and MHC molecules [39, 40]. Therefore, it is quite possible that a TCR will still recognize a peptide if its non-contact amino acid(s) are replaced with biochemically similar or even different amino acid(s) [36, 39, 40]. Further, MHC class I or II molecules can bind to and present a variety of peptides, which differ in amino acid composition [36, 41, 42]. These properties of both MHC molecules and TCR are necessary to process and respond to a vast pool of epitopes originating from a huge number of pathogens and/or antigens [36, 39, 41, 43]. Various studies show that CD4^+^ T cells are more promiscuous than CD8^+^ T cells in peptide epitope recognition [40, 42, 44, 45]. Also the MHC class II peptide binding pocket is more flexible than the MHC class I peptide-binding pocket [42, 45]. A higher level of promiscuity occurs at the CD4^+^ T cell level and flexibility within peptide-MHC II binding allows for greater and less-restrictive CD4^+^ T cell responses. This property is physiologically relevant as CD4^+^ T cell help is required to generate both humoral and effector T cell responses [37, 46]. Studies have also shown that if amino acids on a peptide epitope can be substituted by amino acids with similar biochemical property, it will be still recognized by the same T cell clone [42]. Additionally, some T cell clones could be intrinsically more cross-reactive than others. In outbred human population, the repertoire of peptide presentation and T cell recognition becomes even broader due to co-dominant expression of MHC molecules. Therefore, some individuals may show higher heterologous immunity against certain antigens than other individuals [47, 48].

An interesting aspect of Ad vector induced heterologous immunity emerged in responses to core antigen of HCV. We have previously reported that mice immunized with a recombinant Ad vector expressing whole HCV core protein do not generate core-specific T cell responses due to immunosuppressive effects [29, 30]. In contrast, in the current study we unexpectedly found that an Ad vector without a transgene is able to induce robust core antigen specific humoral and cellular immune responses, due to a high degree of homology between multiple core-derived peptide sequences and various adenovirus proteins. This could be because adenovirus does not encode for a complete sequence of HCV-core but rather exhibits homologies in short peptide sequences, and therefore core-induced immune suppression is not apparent. Interestingly, however, core-stimulated effector cells showed significantly less killing activity than other effectors (Fig 6). The reason is not clear yet.

TLR agonists such as poly I:C engage their receptor and trigger signaling. This results in the upregulation of several costimulatory molecules and cytokines by antigen presenting cells and also T cells, enhancing antigen-specific responses. In our studies, co-administration of poly I:C along with Ad vector boosted the intrinsic heterologous immunity of Ad vector against multiple HCV antigens, further endorsing the Ad vector mediated induction of heterologous immunity against HCV (Figs 2 and 7B).

Humoral immune response is mediated by B cells, which produce antibodies against the pathogen-derived antigens. Antibodies can recognize a linear peptide epitope or a conformational epitope [49, 50]. The cross-reactive antibody response is expected to be contributed by the linear peptide epitopes, which contain identical or biochemically similar amino acids at the Fab’ binding site(s) [50]. Therefore, as HCV and Ad vector proteins show a high level of homology at different sites in their proteins, the probability of cross-reactive Ab-binding epitopes is high and our data suggest that antibodies produced in response to Ad vector immunization also show cross-reactivity with HCV antigens. This was further enhanced upon co-immunization with poly I:C (Fig 2B).

In addition to inducing CD4^+^ and CD8^+^ T cell and antibody responses, our data clearly demonstrate the antiviral potential of heterologous immunity induced by Ad antigens. It can significantly reduce the viral load in Vac-HCV challenged mice, but not in wild-type vaccinia challenged mice (Fig 7A and 7B), and the use of an adjuvant with Ad vector further enhances the cross-reactive antiviral immunity (Fig 7B). Whether cellular and/or humoral arms of cross-reactive immunity confer viral reduction in an advanced model of HCV remains to be determined.

Adenoviruses commonly infect humans, where they have also been shown to exist persistently as normal gut flora [51]. Therefore, we sought to detect HCV-specific cross-reactive immune responses in humans with pre-existing immunity against Ad. Intriguingly, we observed that all of the healthy HCV-naive human donors (n = 19) that were seropositive for anti-Ad IgG (Fig 8A) demonstrated cross-reactive HCV-specific humoral (n = 19) and cellular (n = 17) immunity (Fig 8A and 8B). Interestingly, in the majority of donors, CD4^+^ T cells showed higher induction of IL-10 compared to IFN-γ upon stimulation with different HCV antigens, and only a minority of donors (~17% or 3 out of 17) showed higher levels of IFN-γ expression compared to IL-10 in both CD4^+^ and CD8^+^ T cells. These results represent the existence of two discreet courses of natural cross-reactive cellular immune responses against HCV antigens in humans with pre-existing Ad-specific immunity. The reasons for these two courses are not clear and could be dependent upon HLA type, T cell repertoire and/or environmental factors. However, it can be speculated that exposure of such individuals to HCV may also lead to distinct patterns of immune responses culminating in either acute/self-clearing or chronic infections, with a predisposition towards the development of chronic HCV infection in the majority of cases. These results are intriguing and suggest the potential impact of Ad immunity on the natural course of chronic vs. acute/clearing HCV infection, albeit with a small number of individuals. Prospective immunological studies in a large cohort of individuals will be required to fully predict and correlate Ad immunity to viral clearance vs. persistence upon exposure to HCV.

In conclusion, Ad vector has an intrinsic ability to induce broadly reactive immunity against highly conserved HCV antigens across genotypes, and may have a potential use as therapeutic/prophylactic pan-genotypic vaccine for HCV infection, even without an additional HCV transgene. Rare human and non-human adenoviruses are being developed for Ad vector-based vaccines, to avoid the issues of pre-existing immunity. However, this strategy will not completely rule out any pre-existing inter- and intra-species cross-reactive anti-adenoviral immunity. In fact, it has been shown recently that 12% of human subjects screened are seropositive to chimp Ad virus 3 and 22% are seropositive to a rare human Ad virus 6, in contrast to 38% seropositivity to the prevalent human Ad virus 5 [52]. Further, adenovirus-specific T cell immunity against conserved epitopes has been shown across various human serotypes and also across species [52]. We observed that the homology between HCV epitopes and different adenoviruses spans across a number of adenoviral proteins, and thus it can possibly be extended to a number of uncommon human and non-human adenoviruses.

Heterologous immunity is a double-edged sword, which can modulate the breadth of the T cell repertoire, influencing the memory T cell pool and/or the immune-dominance of a specific epitope, leading to enhanced or diminished immune responses against a pathogen. Such cross-reactive immune responses can explain the spontaneous re-activation of HCV-specific functional immune responses and viral clearance. It can also explain the cases of HCV-specific memory T cells in HCV-naïve individuals. It remains to be established, however, whether and how pre-existing immunity to adenoviruses, and prevalent use of Ad vectors as vaccines for other diseases, shape the course of natural infection with HCV in the human population.

## Materials and Methods

### Sequence Alignment

Fifteen to twenty amino acid long sequences from various HCV antigens of genotype 1a (core, F, NS3, NS4 and NS5) were compared with human adenovirus 5 protein sequences (S1 Table) by sequence alignment using ClustalW software (Online version). Sequence homology was documented as pairwise similarity or homology score, and a heat map was prepared by Microsoft Excel to present the distribution of each HCV antigen peptide homology across the different adenovirus proteins. Pairwise scores in the aligned region are the number of identities between the two sequences, divided by the length of the alignment, and calculated as a percentage, so a score of 25 means 25% homology, 30 means 30% homology between aligned sequences and so on. The number of adenovirus proteins showing various levels of homology (25.00 to 30.00, 30.11–35.00, 35.11–40.00, 40.11–45.00, 45.11–50.00 and >50) was calculated from the heat map of scores, and summarized in Table 1 (see S1–S6 Datasets for details).

### Adenovirus vector

Replication-incompetent human adenovirus 5, with no transgene insert, was amplified and titrated in human embryonic cell line 293A (HEK-293A, source ATCC# CRL 1573) transformed with adenovirus E1 gene (cat# AES 2044, QBiogene Inc., CA, USA) to provide complementarity for virus production. Recombinant adenoviruses (rAd) expressing HCV antigens core (rAd-core), F (rAd-F), NS3 (rAd-NS3), NS4 (rAd-NS4) or NS5 (rAd-NS5) were prepared and reported earlier by us [17, 28, 31].

### DNA Purification and PCR Amplification

DNA was purified from Ad, rAd-core, rAd-NS3, rAd-NS4, rAd-NS5a and rAd-NS5b vector stocks. Briefly, 1x10^8^ pfu of each vector was taken in individual tubes and DNA was prepared by using High-Pure Viral Nucleic Acid KitR (cat.# 11 858 874 001, Roche Applied Bio). PCR reaction was set up with 10 μl template DNA obtained from the above preparation using 50 μl total reaction volume consisting of 1x PCR buffer, 10 μM dNTP, 25 μM of each primer (forward and reverse, listed in S2 Table) and 1.25 unit of Taq polymerase. PCR tubes containing reaction mixtures were incubated in a thermo-cycler with initial denaturation at 92°C and 40 amplification cycles (92°C: 30 Sec, 50–55°C: 30 Sec, 68°C: 60 Sec). PCR amplification products were run on 1% agarose gel at 80 volts to resolve amplification products along with 1 KB sized Quick Load DNA Ladder (Cat.# N0468S, NEB Biolab, Germany).

### Adjuvants

Toll-like receptor-3 agonist poly I:C (Cat.# P1530, Sigma-Aldrich, St. Louis, MO, USA) was used as an adjuvant for immunization with Ad vector.

### Mice immunizations

Six to seven week-old female C57Bl/6 mice were purchased from Charles River Laboratory (Charles River, Canada) and immunized twice intramuscularly (at 14-day intervals) using an optimized dose (2x10^7^ PFU/mouse) of Ad, in presence or absence of adjuvant (Poly I:C, 20 μg/mouse). Mice were euthanized 8 days after the second immunization and various tissue samples (e.g. spleens, inguinal lymph nodes, ovaries, serum etc.) were collected.

### T cell proliferation assay

Eight days after the second immunization, mice were euthanized, and spleens and/or inguinal lymph nodes were collected. The spleens were pooled from replicates and ground to single cell suspensions and filtered through a Falcon 100 μm nylon cell strainer. The cells were resuspended in 2 ml of media and passed through an equilibrated nylon wool column. The column was washed after 45 min of incubation at 37°C and the flow through contained the splenic T cells. These T cells were used in the experiments (~90% CD3^+^ T cells). Lymph nodes were also ground into single cell suspensions and used in the assays. Proliferative responses were measured in triplicate cultures in 96-well flat-bottomed microtiter plates. A total of 4×10^5^ T cells from immunized mice and 4×10^5^ APCs (spleen cells from unimmunized mice irradiated with 3000 rads) were mixed with different HCV-derived proteins (core = c22-3, NS3 = c33c; NS4 = c100-3; NS5 = NS5 SOD, polyprotein c25 = core+NS3+NS4; polyprotein c200 = NS3+NS4 or control protein = rhSOD, kindly provided by Chiron/Novartis) or synthetic HCV-derived peptides (see S1–S6 Datasets) at different concentrations as described in the Fig legends. T cell proliferation was assessed by a radioactive ^3^H-thymidine incorporation assay. Detailed methodologies for T cell proliferation assays have been reported previously by us [32].

### **IFN-**γ **Cytokine ELISA**

Levels of IFN-γ were assessed in culture supernatants collected from T cell proliferation assays using mouse IFN-γ ELISA kits supplied by eBiosciences (Cat.# 88–7314, eBiosciences Inc. San Diego, USA). ELISA was performed according to the manufacturer’s instructions. Plates were read and data were analyzed in FluoStar ELISA reader (BMG Labtech GmbH, Ortenberg, Germany). Cytokine concentration per ml of culture supernatant was determined by multiplying the calculated concentration with the dilution factor. The averages of these concentrations (pg/ml) from duplicate wells were plotted in the graphs shown here.

### Antibody ELISA

Serum was prepared from the blood of immunized mice and stored at -20°C until required. HCV antigen-specific cross-reactive IgG antibodies in Ad vector immunized mice, were measured in 96-well plates coated overnight at 4°C with HCV antigens (core, NS3, NS4 or NS5) at 1 μg/ml in 1xPBS. The next day, after blocking with 1% BSA at room temperature for 1 hour, serial dilutions of serum samples were added to the 96-well plate in 2–3 replicates and incubated again at room temperature for 2 hours. After application of serum, anti-mouse IgG labeled with alkaline phosphatase (AP) (Cat.# 1031–04, Southern Biotech, Alabama, USA) was added and plates were incubated for 1 hour. Color was developed by adding PNPP substrate (Cat.# 0201–01, Southern Biotech, Alabama, USA). Plates were washed with 1xPBST (1xPBS with 0.1% Tween-20) after each incubation step. Absorbance was read using FluoStar Optima ELISA Reader (BMG Labtech GmbH, Ortenberg, Germany), and OD values from HCV antigen-coated plates, corrected for background from OD values in SOD-coated plates, were plotted in the graphs shown here.

### Flow cytometry

Mouse splenocytes were cultured with 5 μg/ml of HCV protein antigens or peptides for 5 days in RPMI-1640 media supplemented with 10% fetal bovine serum. On the fifth day, cells were harvested and counted, and 1x10^6^ cells per group were stained for T cell markers. To perform intracellular cytokine staining, splenocytes cultured for 5 days with HCV antigens were treated with ionomycin (1 μg/ml), phorbol 12-myristate 13-acetate or PMA (50 ng/ml) and brefeldin A (1.5 μg/ml, Cat.# 00–4506) for 5 hours and subsequently stained by fluorochrome labeled antibodies (purchased from eBiosciences Inc. San Diego, USA) for extracellular lineage markers: CD3 (PE Cy7, Cat.# 25–0031), CD4 (APC, Cat.# 17–0441) and CD8a (APC efluor 780, Cat.# 47–0081); and intracellular cytokines: IFN-γ (PE, Cat.# 12–7311) and IL-10 (FITC, Cat.# 11–7101). For T cell proliferation using the CFSE dilution assay, splenocytes were enriched for T cells using nylon wool column and stained with 1 μM CFSE (Cat.# 21888, Sigma-Aldrich, St. Louis, MO, USA) in 1 ml of cell suspension for 7–10 minutes at room temperature. These CFSE stained cells were washed in 1x PBS containing 10% fetal bovine serum, counted and plated in 24-well plate with 5 μg/ml of HCV core, NS3, NS4 or NS5 antigen or their representative peptides; and equal number of γ-irradiated syngeneic splenocytes as APCs. These cells were cultured for four days and stained for CD3^+^, CD4^+^ and CD8^+^ T cell markers. T cells stained with isotype control antibodies with corresponding fluorochromes (PE Cy7: cat.# 25–4888, APC: cat.# 400511, APC eFluor 780: cat.# 47–4321, PE: 400507 and FITC: cat.# 400905; purchased from eBiosciences, Inc. San Diego, USA) were used to exclude non-specific binding. All stained cells were run in a BD FACS Canto (BD Biosciences, San Jose, CA, USA), and data were analyzed using FCS Express 4.0 software (De Novo Software, Glendale, CA, USA).

### T cell cytotoxicity assay

Splenocytes harvested from Ad vector immunized mice were stimulated *in vitro* with the HCV protein antigens core, NS3, NS4, NS5 or polyprotein at 5 μg/ml for 4 days. The target EL4 cells were incubated with corresponding HCV peptides (core peptides #: 2, 14, 17, 25, 27, 28, 32; NS3 peptides #: 8, 10; NS4 peptides #: 3, 4, 8; NS5a peptides #: 1, 2, 16, 20, and NS5b peptides #: 5, 19, 23, 39; or All: a mixture of the above peptides from core, NS3, NS4 and NS5) overnight at 37°C. Next day, peptides loaded EL4 cells were cultured with effectors at 10:1 (effectors: target) ratio for 4–5 hours. CFSE-labeled live targets were quantified by flow cytometry and subtracted from background CFSE-labeled targets to get the numbers of killed targets. Empty (no peptide loaded) EL4 targets were used as a negative control.

### Chimeric Vac-HCV Challenge

Eight days after the second immunization with Ad vector, Ad vector + poly I:C or PBS, mice were challenged with 1x10^7^ PFU of Vac-HCV chimeric virus (Vac-C/NS2/NS3 including core, NS2 and NS3 antigens of HCV or Vac-NS3/NS4/NS5 including NS3, NS4 and NS5 antigens of HCV, kindly provided by Dr. Alfred Prince, NY, USA), or wild-type vaccinia (WT-Vac, not containing HCV antigens) virus intraperitoneally. Five days after virus challenge, mice were euthanized and ovaries were removed, homogenized and freeze-thawed three times in 1x PBS. Homogenized samples were stored at -80°C until used for viral titer.

### Vac-HCV Titration by Plaque Assay

Serially diluted samples of ovary homogenates were added in duplicate wells in 6-well plates containing 80% confluent monolayers of mouse lymphoma cell line TK-1 (ATCC # CRL 2396) and incubated for 90 minutes. Subsequently, unbound virus was removed and fresh 1x DMEM medium (Cat.# 11995, Gibco by Invitrogen, NY, USA) supplemented with 3% FBS, was added, and plates were incubated for 48 hours. At this time, the medium was removed, and plaques were fixed by using 10% formaldehyde (Cat.# BP531-500), Fisher Scientific, NJ, USA) at room temperature for 30 minutes. Plates were washed with PBS and the monolayers were stained for 30 minutes with 0.5% crystal violet (Cat.# C3886, Sigma-Aldrich Company, MO, USA), followed by further washing. Plaques were counted, averaged and multiplied with the dilution factor to determine the viral load/mouse. Data are presented as % reduction of viral load, and calculated by the formula: [(Mean viral titer in control group-Titer in immunized group)/Mean viral titer in control group] X 100. Control groups had viral titers in the range of 10^7^−10^8^ pfu/mouse (Vac-C/NS3) and 10^8^−10^9^ pfu/mouse (Vac-NS3/4/5).

### Human blood donors, Plasma and PBMCs

Peripheral blood samples were collected from healthy human donors of both sexes with no known history of HCV infection. Collected blood was processed immediately for PBMCs isolation using Ficoll gradient separation. The upper plasma layer was frozen in aliquots for IgG ELISA, and the middle PBMCs layer was collected, washed and frozen until use.

### Human IgG antibody ELISA

Anti-Ad IgG (hexon-specific) antibodies were detected and evaluated in the plasma of each donor using IBL Adenovirus IgG ELISA Kit (Cat# IB79202, IBL-America Inc. Minneapolis, MN, USA). Plasma samples were assayed at 1:100 and 1:400 dilutions in duplicates according to the manufacturer’s procedure. HCV antigens-specific cross-reactive IgGs were determined by ELISA. Briefly, ELISA plates were coated with recombinant HCV antigens: core NS3, NS4, NS5 or control protein SOD (superoxide dismutase) at 1 μg/ml overnight at 4°C followed by blocking with 1% BSA (bovine serum albumin) for 1 hour. Plasma samples (1:100 to 1:400) were added in triplicate and incubated for 2 hours at room temperature. Binding of antigen-specific Human IgG was detected by adding anti-human IgG labeled with alkaline phosphatase (AP) (Cat# 2040–04, Southern Biotech, Alabama, USA) for 1 hour, followed by addition of PNPP substrate (Cat#0201–01, Southern Biotech, Alabama, USA) for 30 minutes. Plates were washed with 1x PBST (1x PBS with 0.1% Tween-20) after each incubation step. Absorbance (OD) was measured at 405 nm, and corrected for background by subtracting the OD values obtained in control SOD-coated plates. Absorbance was measured using a FluoStar Optima ELISA Reader (BMG Labtech GmbH, Ortenberg, Germany). For statistical analyses, OD values from anti-Ad IgG ELISAs were arranged in increasing order and correlated with corresponding OD values from anti-HCV IgG. Correlation coefficients were calculated by Spearman's rank correlation.

### *Ex vivo* stimulation of human PBMCs and intracellular cytokine staining

Frozen PBMCs were thawed and washed with warm PBS twice, counted and cultured in AIM-V serum free medium (Cat# 12055–083, Gibco by Life Technologies, NY, USA) in 96-well plate at 0.5x10^6^ cells/100 μl/well for 2 hours. HCV pooled synthetic peptides (core peptides # 5, 14, 16, 17 & 27; NS3 peptides # 2, 5, 6, 8 &10; NS4 peptides # 4, 8, 9, 13, 16 or NS5 peptides # NS5a: 6, 24 + NS5b: 5, 19, 27) or individual proteins (core, NS3, NS4 or NS5) were added to the plates at 5 μg/ml concentration. The peptides were selected on the basis of their high sequence homologies with Ad proteins, and detection of cross-reactive responses in mouse experiments. Phyto-hemagglutinin (PHA, 1 μg/ml) and media-treated cells were considered as positive and negative controls, respectively. After three days of incubation, cells were treated with 1.5 μg/ml brefeldin A (Cat.# 00–4506, eBiosciences Inc., San Diego, CA, USA) for 5 hours, and stained for extracellular lineage markers using fluorochrome-labeled antibodies (purchased from eBiosciences Inc. San Diego, USA): CD3 (eFluor 450, Cat.# 48–0036), CD4 (FITC, Cat.# 11–0049) and CD8a (PE, Cat.# 12–0089); and for intracellular cytokines: IFN-γ (APC, Cat.# 17–7319) and IL-10 (PE-Cyanine 7, Cat.# 25–7108). Stained cells were measured in a BD FACS Canto, and data were analyzed using FACS Diva software. The CD3^+^ T cell population was gated for CD4^+^ and CD8^+^ subsets and evaluated separately for IFN-γ and IL-10 expression. PBMCs stained with isotype antibodies with corresponding fluorochromes (Cat.# 48–4714, 11–4714, 12–4714, 17–4714 and 25–4301; purchased from eBiosciences, Inc. San Diego, USA) were used to exclude non-specific binding. Percent increase in cytokine expression was calculated by the formula: [(Antigen treated cells- media control)/ media control] x 100. The percentage of cells positive for IFN-γ, IL-10 and IFN-γ/IL-10 in the media groups ranged from 0.2–10% in different donors.

### Statistical Analysis

Data were analyzed by Graph-pad Prism software (Graph-pad Software Inc., CA, USA). Two tailed student *t*-test was used to determine the significant difference between two groups, and *P*-values less than 0.05 (<0.05) were considered to be statistically significant.

### Ethics Statement

All animal experiments were approved by University of Alberta Animal Care and Use Committee (protocol number AUP 212 till June 30, 2016) in accordance with the Canadian Council of Animal Care guidelines. The written informed consent was obtained from all adult blood donors. All procedures related to human tissue were approved (number 3983 till Nov 9, 2016) and were in accordance with the University of Alberta Human Research Ethics Board (HREB).

## Supporting Information

S1 Dataset List of HCV core protein derived peptides and number of Ad proteins showing homology. (TIF)

S2 Dataset List of HCV F protein derived peptides and number of Ad proteins showing homology. (TIF)

S3 Dataset List of HCV NS3 protein derived peptides and number of Ad proteins showing homology. (TIF)

S4 Dataset List of HCV NS4 protein derived peptides and number of Ad proteins showing homology. (TIF)

S5 Dataset List of HCV NS5a protein derived peptides and number of Ad proteins showing homology. (TIF)

S6 Dataset List of HCV NS5b protein derived peptides and number of Ad proteins showing homology. (TIF)

S1 Fig Identification of cross-reactive T cell immune responses (proliferation and IFN-γ production) against individual synthetic peptides derived from HCV antigens in Ad vector immunized mice. Cross-reactive immune responses against core, F, NS3, NS4, NS5a and NS5b derived synthetic peptides in Ad vector immunized mice were evaluated, to characterize and identify the domains of cross-reactivity in various HCV antigens with respect to amino acid sequences. Proliferative and IFN-γ cytokine responses were determined by procedures as described in materials and methods section. Many of the HCV core, F, NS3, NS5a and NS5b peptides were able to induce T cell proliferation *ex vivo*, which also translated into production of IFN-γ. These peptides had high amino acid sequence homology and multiple high scoring regions with the different Ad proteins. However, some peptides, which showed high homology with respect to high score (>35) and number of homologous regions in Ad proteins, did not show cross-reactive responses in mice immunized with Ad vector (S1A–S1F Fig and S1–S6 Datasets). Response to HCV proteins: core, NS3, NS4 and NS5 are also presented in graphs (S1A–S1F Fig). Data are presented as mean + standard deviation of triplicate (counts per minutes, CPM) or duplicate wells (cytokine concentration), and are representative of two independent experiments.
(TIF)

S2 Fig Dose dependent cross-reactive proliferation of (A) spleen and (B) lymph node T cells obtained from mice immunized with Ad vector in presence or absence of poly I:C adjuvant upon stimulation with HCV core, NS3 and NS5 protein derived peptide pool. (TIF)

S1 Table Description of adenoviral (Ad) proteins, which were compared to determine homologies with HCV proteins derived peptide epitopes. (DOCX)

S2 Table PCR primers used to detect presence of HCV genes in adenoviral vector stock. (DOCX)
