# Supplementary figures and images for: Heterologous Immunity between Adenoviruses and Hepatitis C Virus: A New Paradigm in HCV Immunity and Vaccines
Source: PLoS One. 2016 Jan 11;11(1):e0146404. doi: 10.1371/journal.pone.0146404 (PMC4709057; doi:10.1371/journal.pone.0146404)

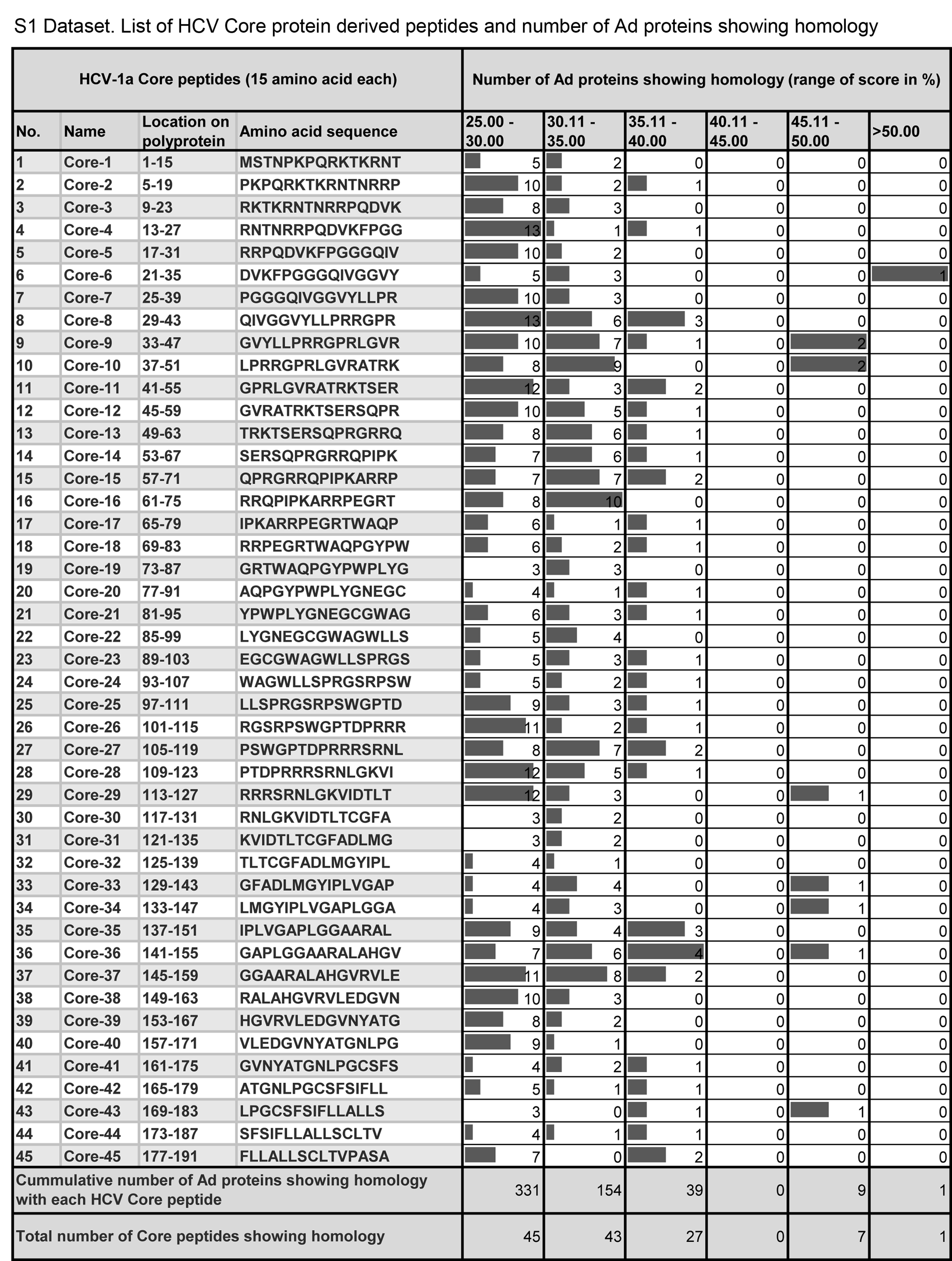

Supplement: S1 Dataset — (TIF) [file pone.0146404.s001.tif]

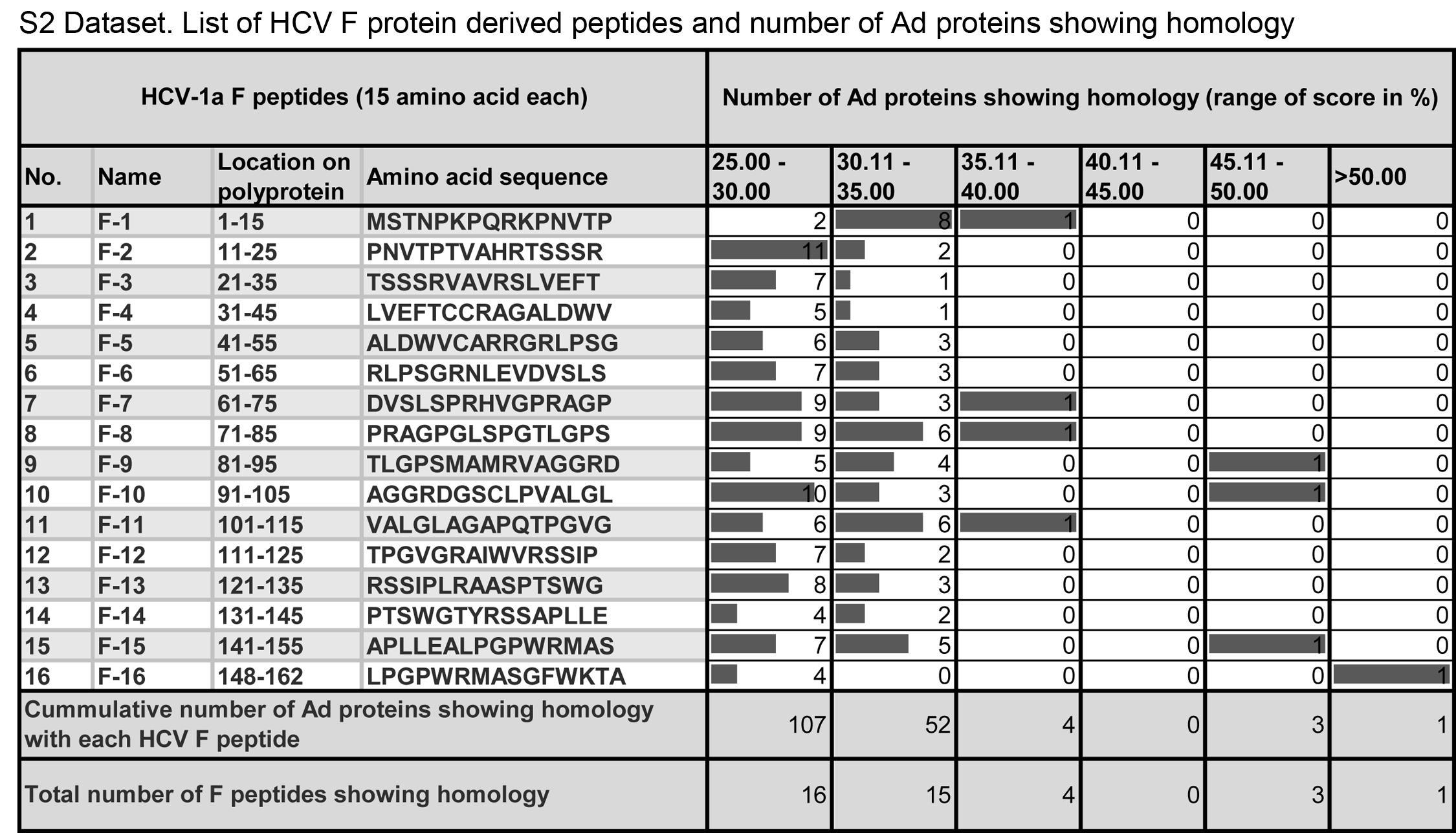

Supplement: S2 Dataset — (TIF) [file pone.0146404.s002.tif]

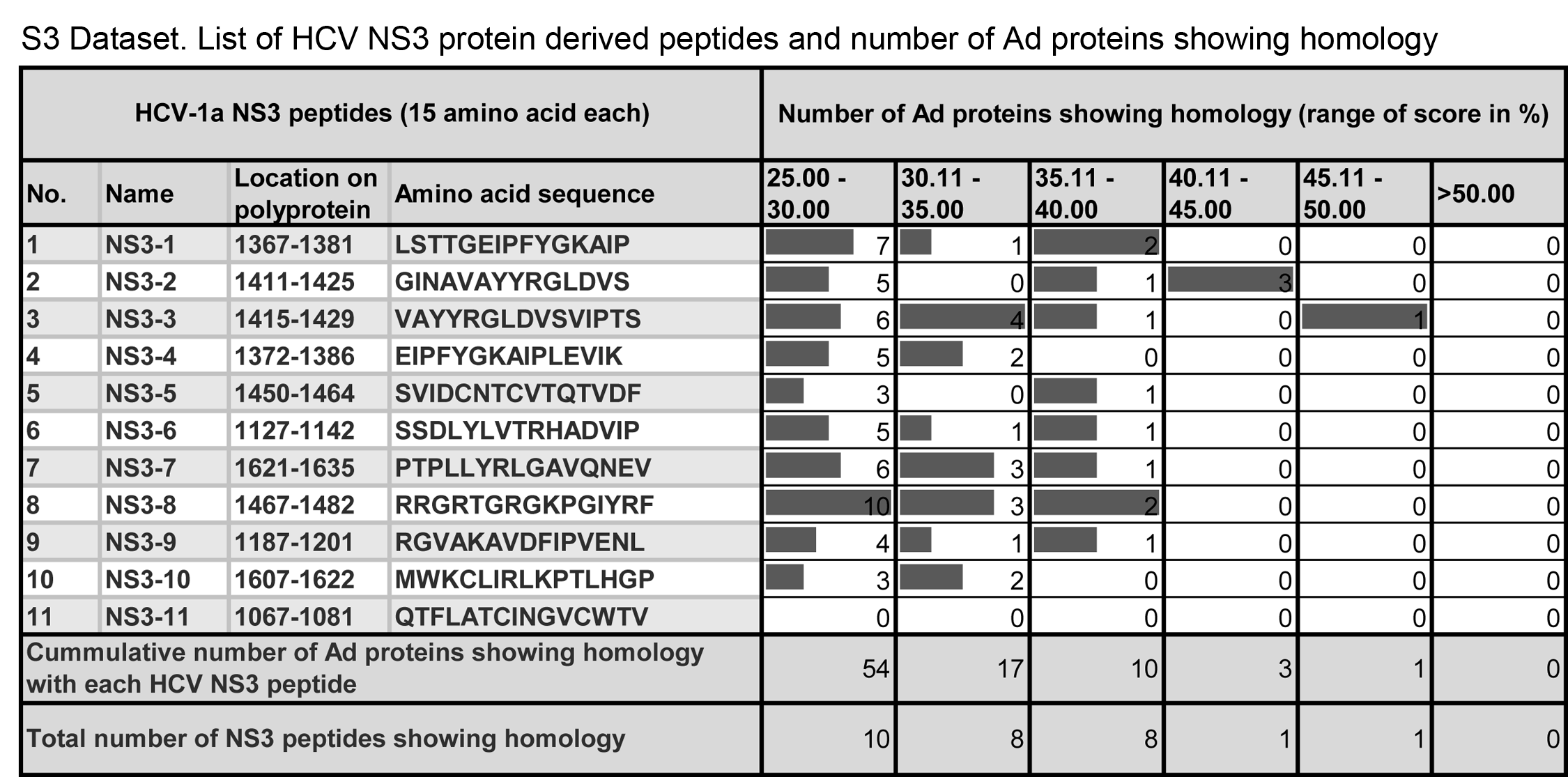

Supplement: S3 Dataset — (TIF) [file pone.0146404.s003.tif]

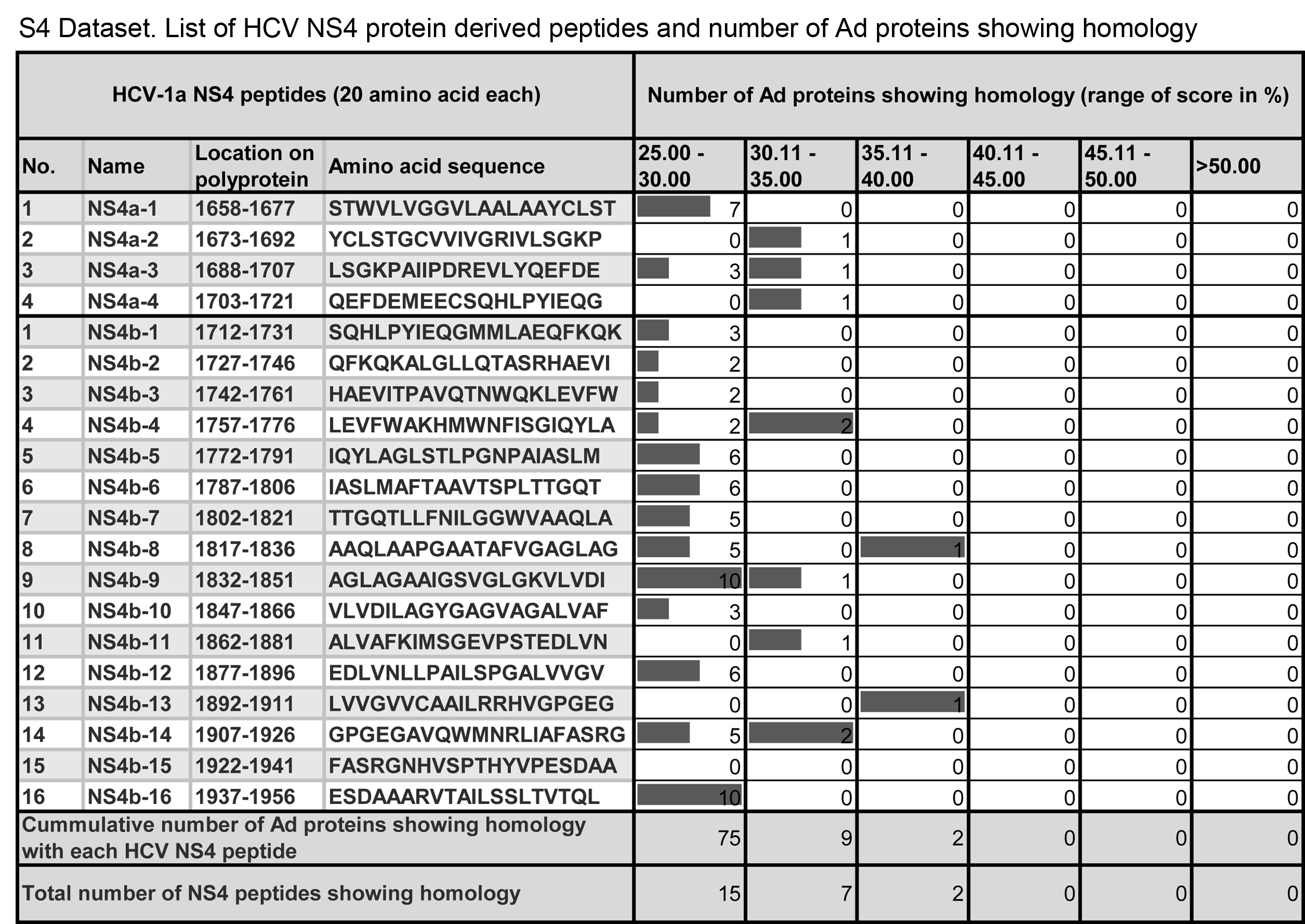

Supplement: S4 Dataset — (TIF) [file pone.0146404.s004.tif]

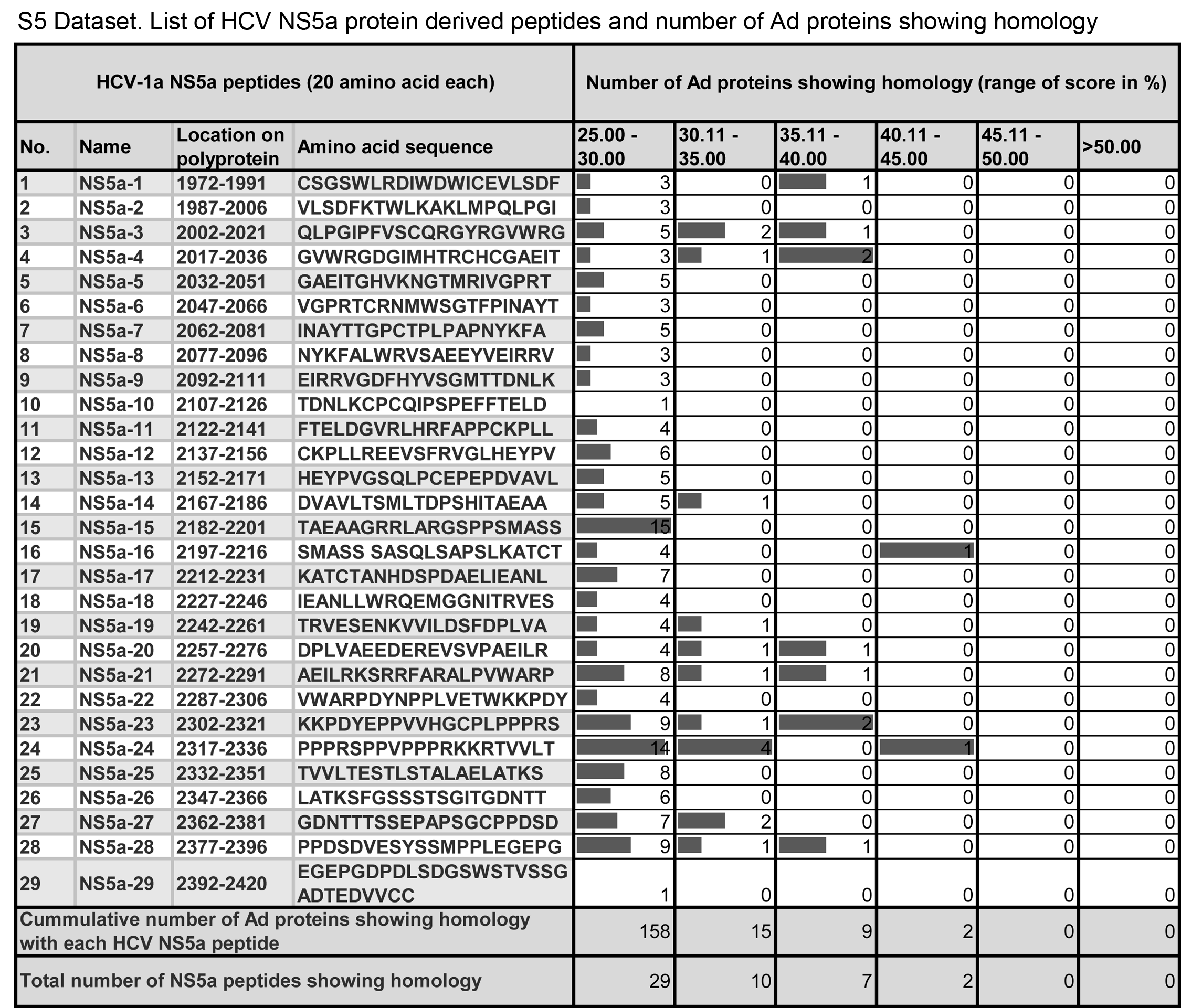

Supplement: S5 Dataset — (TIF) [file pone.0146404.s005.tif]

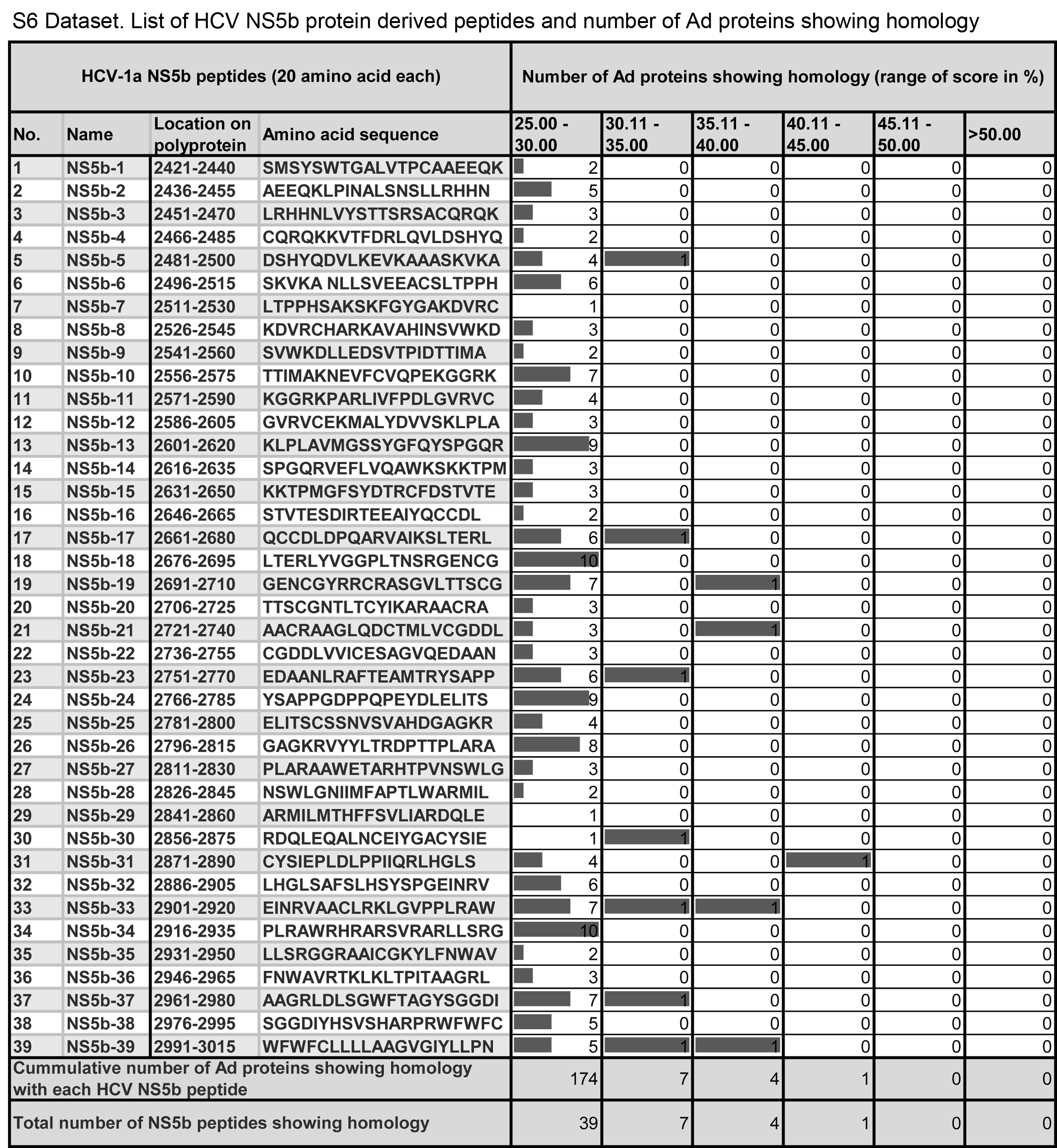

Supplement: S6 Dataset — (TIF) [file pone.0146404.s006.tif]

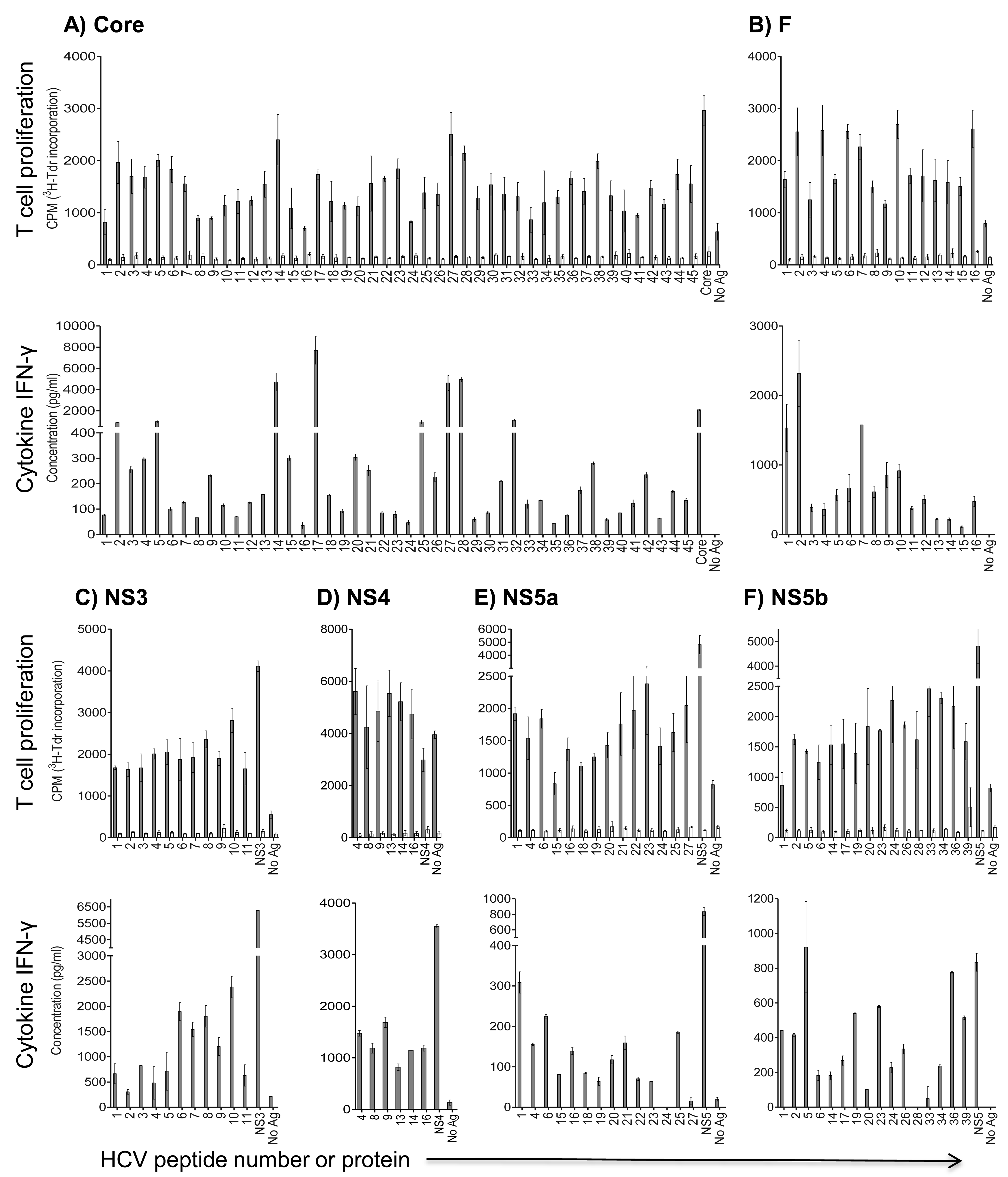

Supplement: S1 Fig — Cross-reactive immune responses against core, F, NS3, NS4, NS5a and NS5b derived synthetic peptides in Ad vector immunized mice were evaluated, to characterize and identify the domains of cross-reactivity in various HCV antigens with respect to amino acid sequences. Proliferative and IFN-γ cytokine responses were determined by procedures as described in materials and methods section. Many of the HCV core, F, NS3, NS5a and NS5b peptides were able to induce T cell proliferation ex vivo, which also translated into production of IFN-γ. These peptides had high amino acid sequence homology and multiple high scoring regions with the different Ad proteins. However, some peptides, which showed high homology with respect to high score (>35) and number of homologous regions in Ad proteins, did not show cross-reactive responses in mice immunized with Ad vector (S1A–S1F Fig and S1–S6 Datasets). Response to HCV proteins: core, NS3, NS4 and NS5 are also presented in graphs (S1A–S1F Fig). Data are presented as mean + standard deviation of triplicate (counts per minutes, CPM) or duplicate wells (cytokine concentration), and are representative of two independent experiments. (TIF) [file pone.0146404.s007.tif]

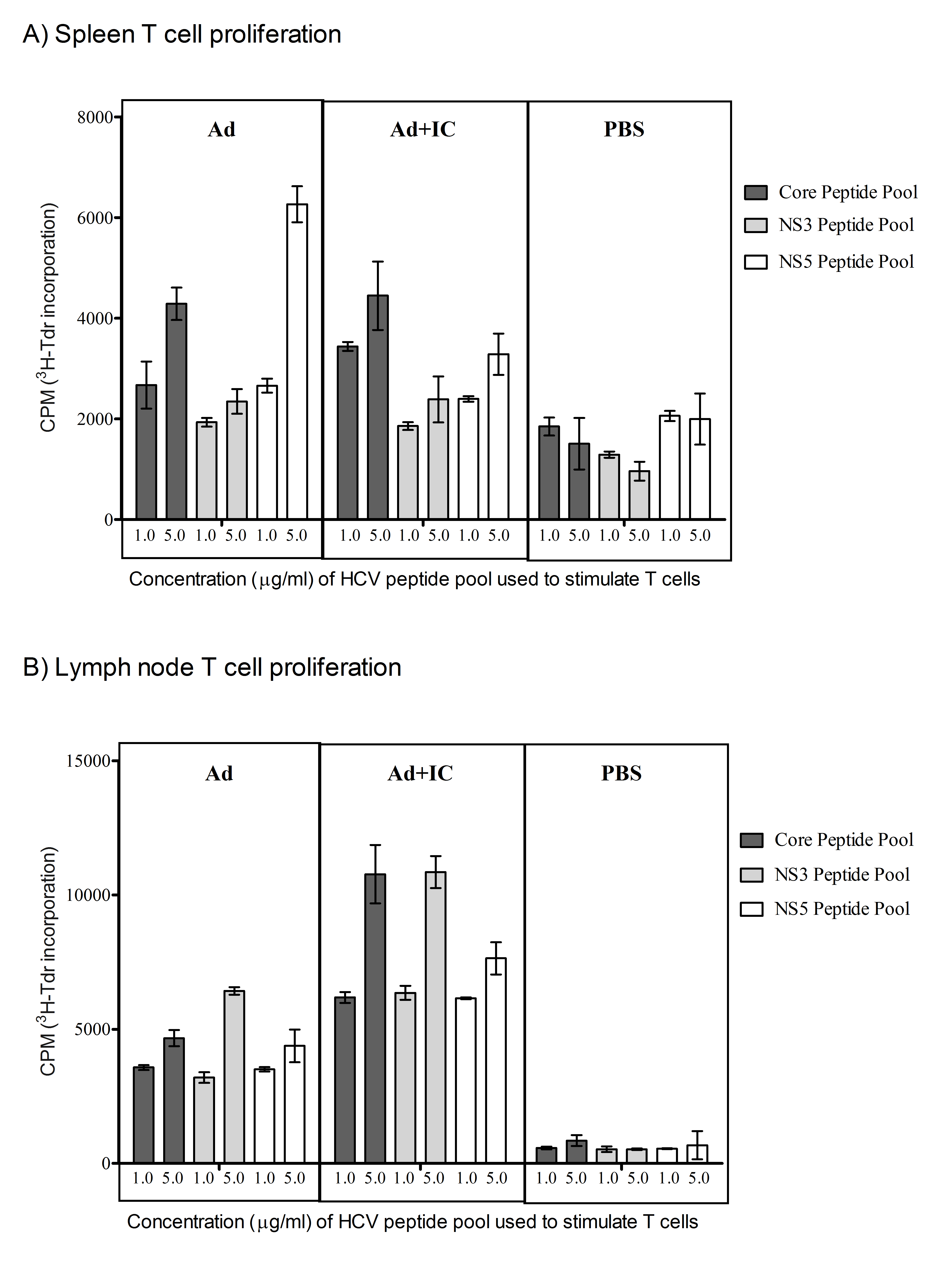

Supplement: S2 Fig — (TIF) [file pone.0146404.s008.tif]
