# Supplementary material for: Heterologous Immunity between Adenoviruses and Hepatitis C Virus: A New Paradigm in HCV Immunity and Vaccines
Source: PLoS One. 2016 Jan 11;11(1):e0146404. doi: 10.1371/journal.pone.0146404 (PMC4709057; doi:10.1371/journal.pone.0146404)
Supplement: S1 Table — (DOCX) [file pone.0146404.s009.docx]

| **No.** | **Human Ad5 protein name** | **Short name** | **GI number** | **Accession no.** | **Transc-ription unit** |
| --- | --- | --- | --- | --- | --- |
| 1 | E2A, DNA Binding Protein | DBP | 58177697 | AAW65516 | E2 |
| 2 | E2B, DNA Polymerase | Pol | 58177697 | AP_000202 | E2 |
| 3 | E2B, Terminal Protein Precursor | pTP | 209846 | AAW65504 | E2 |
| 4 | 34k, Control Protein | 34K | 58177724 | AAW65531.1 | E4 |
| 5 | ORF1, Control Protein | ORF1 | 56160565 | AP_000232.1 | E4 |
| 6 | ORF2, Control protein | ORF2 | 56160564 | AP_000231.1 | E4 |
| 7 | ORF3, Control protein | ORF3 | 56160563 | AP_000230.1 | E4 |
| 8 | ORF4, Control protein | ORF4 | 56160562 | AP_000229.1 | E4 |
| 9 | ORF6/7, Control Protein | ORF6/7 | 56160560 | AP_000227.1 | E4 |
| 10 | 13.6k protein | 13.6K | 58177696 | AAW65503.1 | L1 |
| 11 | 52k, Encapsidation Protein | 52K | 58177700 | AAW65507.1 | L1 |
| 12 | pIIIa, Capsid Protein precursor | pIIIa | 58177701 | AAW65508.1 | L1 |
| 13 | III, Penton Base | Penton | 5817770 | AAW65509 | L2 |
| 14 | pVII, Core Protein Precursor | pVII | 209845 | AAA96408.1 | L2 |
| 15 | V, Core Protein | V | 209846 | AP_000208 | L2 |
| 16 | pX, Core Protein Precursor | pX | 58177705 | AP_000209 | L2 |
| 17 | pVI, Core Protein Precursor | pVI | 209848 | AP_000210 | L3 |
| 18 | II, Hexon (Capsid) protein | Hexon | 157879600 | 1P30_A | L3 |
| 19 | Protease | Protease | 58177708 | AP_000212 | L3 |
| 20 | 100k, Hexon Assembly Protein | 100k | 58177710 | AAW65517.1 | L4 |
| 21 | 22k Protein | 22k | 58177711 | AAW65518.1 | L4 |
| 22 | 33k protein | 33K | 58177712 | AAW65519.1 | L4 |
| 23 | pVIII, Capsid Protein precursor | pVIII | 454806 | AP_000217 | L4 |
| 24 | IV, Fiber (Capsid) Protein | Fiber | 209931 | AP_000226 | L5 |
| 25 | Encapsidation Protein IVa2 | IVa2 | 56160534 | AP_000201.1 | IVa2 |
| 26 | Capsid Protein IX | IX | 56160533 | AP_000200.1 | IX |
| 27 | Protein U | U | 158536737 | ABW72885.1 | U |

**S1 Table. Description of adenoviral (Ad) proteins which were compared to determine homologies with HCV proteins derived peptide epitopes**
