# Supplementary material for: Heterologous Immunity between Adenoviruses and Hepatitis C Virus: A New Paradigm in HCV Immunity and Vaccines
Source: PLoS One. 2016 Jan 11;11(1):e0146404. doi: 10.1371/journal.pone.0146404 (PMC4709057; doi:10.1371/journal.pone.0146404)
Supplement: S2 Table — (DOCX) [file pone.0146404.s010.docx]

| **Gene** | **Primer sequence** | **PCR product size (bp)** |
| --- | --- | --- |
| Core | Forward: 5′-CGG GAT CCA TGA GCA CGA ATC CTA AAC C-3′  Reverse: 5′-CGG GAT CCT AGG CTG AAG CGG GCA CAG-3′ | 573 |
| F | Forward: 5′-GAA GAT CTA TGC CAA ACG TAA CAC CAA CCG TC-3′  Reverse: 5′-GAA GAT CTC ACG CCG TCT TCC AGA ACC CGG A-3′ | 486 |
| NS3 | Forward: 5′-CGG GAT CCA TGG CGC CCA TCA CGG CGT AC-3′  Reverse: 5′-CGG GAT CCT ACG TGA CGA CCT CCA GGT C-3′ | 1893 |
| NS4 | Forward: 5′- GAA GAT CTA TGA GCA CCT GGG TGC TCG TT-3′  Reverse: 5′- GAA GAT CTC AGC ATG GAG TGG TAC ACT C-3′ | 943 |
| NS5a | Forward: 5′- GAA GAT CTA TGT CCG GTT CCT GGC TAA GG-3′  Reverse: 5′- GAA GAT CTC AGC AGC ACA CGA CAT CTT C-3′ | 1343 |
| NS5b | Forward: 5′- CGG GAT CCA TGT CAA TGT CTT ATT CCT GG-3′  Reverse: 5′- CGG GAT CCT CAT CGG TTG GGG AGG AGG TA-3′ | 1776 |

**S2 Table. PCR primers used to detect presence of HCV genes in adenoviral vector stock**
